# Production of offspring via the transplantation of frozen germ cells from Tokyo bitterling, a fish on the brink of extinction

**DOI:** 10.1038/s41598-025-24449-y

**Published:** 2025-11-19

**Authors:** Kohju Yamakawa, Kiwamu Kawaguchi, Goro Yoshizaki

**Affiliations:** 1 Department of Marine Biosciences, Tokyo University of Marine Science and Technology, 4-5-7 Konan Minato-Ku, Tokyo, 108-8477 Japan; 2 IDEA Consultants, Inc, Kanagawa, Japan; 3 Institute for Aquaculture Technology, Tokyo University of Marine Science and Technology, Tokyo, Japan

**Keywords:** Ecology, Ecology, Zoology

## Abstract

Many freshwater fish face extinctions due to habitat destruction and overfishing. Bitterlings, which lay their eggs in the gill chambers of freshwater mussels, are in steep decline worldwide, along with mussels, owing to the concreting and siltation of riverbeds. In particular, Tokyo bitterling is listed as “endangered” on the Red List and is on the verge of extinction. Although in situ and ex situ conservation efforts are underway, their effectiveness is limited. Here, we developed a method for long-term preservation of genetic resources by combining germ cell cryopreservation and transplantation. We transplanted Tokyo bitterling germ cells stored in liquid nitrogen into oily bitterling recipients lacking endogenous germ cells due to *dnd* knockdown, and the recipients matured and repeatedly produced many eggs and sperm. The resulting offspring had the Tokyo bitterling genotype and morphology. This study shows that even extinct species can be revived if their germ cell bank is available.

**Supplementary Information:**

The online version contains supplementary material available at 10.1038/s41598-025-24449-y.

## Introduction

Although freshwater areas cover less than 1% of the Earth’s surface, these regions are habitats for one-third of all vertebrates^1^ and are known for their high biodiversity. Freshwater fish are so diverse that up to 51% of fish species live in this area, despite their limited environment. However, due to human activities, freshwater biodiversity is declining at an unprecedented rate compared with terrestrial and saltwater biodiversity^2^. Sayer et al*.* (2025), who systematically assessed the global extinction risk of freshwater fauna, reported that 25% of freshwater decapod crustaceans, fishes and odonates were considered threatened with extinction^3^.

Bitterlings, which belong to the subfamily *Acheilognathinae,* are widely distributed throughout Eurasia, with many species in East Asia. However, many of these species are declining and are threatened with extinction (https://www.iucnredlist.org/). This decline is due primarily to the reproductive characteristics of bitterlings. Female bitterlings use ovipositors to lay their eggs in the gill chambers of freshwater mussels of the genus *Unionoida*. After males release sperm near the inhalation siphon of the mussel, the eggs and sperm are fertilized inside the mussel. The fertilized eggs then grow, and larvae emerge into the environment when yolk absorption is complete^4^. In recent years, however, the number of freshwater mussels has declined substantially because of the accumulation of silt on riverbeds, the concreting of riverbeds, and drought, which has accelerated the decline in the number of bitterlings^5–7^. The Tokyo bitterling (*Pseudorhodeus tanago*) is one of the most endangered of the 16 species of native bitterlings distributed in Japan^7^. This species is designated “Endangered” in the IUCN RED LIST and is strictly protected as a "National Natural Treasure" by law, which prohibits its capture, keeping, and transfer (Japanese Ministry of the Environment).

This species used to be a localized inhabitant in the whole Kanto region of Japan but is now only known to survive in less than 10 small waterways between rice fields and ponds^5,6,8,9^. In addition to the decline in the number of mussels used as spawning substrates, another reason for this decline is that these fish spawn an extremely small number of eggs (1–14 eggs/spawning) at a time^8,10^. Conservation measures such as habitat restoration^11,12^ and ex situ conservation by captive breeding^13^ are already in place (https://www.bdcchiba.jp/miyakotanago) to protect these fish. Habitat restoration is a fundamental solution but involves various risks of environmental destruction due to disasters, requires considerable time and cost, and requires periodical maintenance. In contrast, although strain preservation by captive breeding allows stable breeding, this method has many potential problems such as mass mortality due to accidents in rearing facilities and decreased genetic diversity. Indeed, long-term isolation of populations within and outside their original distribution range, as well as reduced genetic diversity and inbreeding due to population size decline, has been proposed to increase the risk of extinction^14–17^. Therefore, low-cost, reliable, and stable technologies for the preservation of Tokyo bitterling genetic resources are urgently needed.

The cryopreservation of gametes or embryos is an alternative to current preservation methods for the genetic resources of endangered species. In many fish species, sperm cryopreservation has been established^18^. However, fish eggs and embryos are extremely difficult to cryopreserve because of their very large size, high yolk content, and low membrane permeability^19–22^.

In recent years, surrogate broodstock technology using germ cell transplantation has attracted attention as a method for preserving genetic resources^23,24^. In this technique, undifferentiated germ cells isolated from donor testes^25,26^ or ovaries^27,28^ are transplanted into the abdominal cavity of recipient larvae. Subsequently, the donor germ cells migrate into the genital ridges of the recipient, where they proliferate and differentiate, allowing the mature recipient to produce donor-derived gametes. This technique has been used successfully with cryopreserved donor testes and ovaries of rainbow trout^29,30^. Following these studies, the technique has been applied to several experimental model fishes and farmed fishes^23,24^. However, there are no examples of this method actually being applied to fish species that have been pushed to the brink of extinction. If these techniques could be applied to Tokyo bitterling, the preservation of its genetic resources would theoretically be semipermanent, thus buying time for habitat restoration.

Therefore, in this study, we aimed to maintain stable long-term preservation of the genetic resources of the endangered Tokyo bitterling and conducted development of a method for cryopreservation of undifferentiated germ cells of this species and establishment of a technology to produce Tokyo bitterling from frozen germ cells using oily bitterling (*Tanakia limbata*), as a recipient. The oily bitterling is classified as “Least Concern” on the IUCN Red List, and its population is far more abundant than that of the endangered Tokyo bitterling. Importantly, the oily bitterling belongs to a genus relatively close to that of the Tokyo bitterling, making it a genetically suitable surrogate. In surrogate reproduction of bitterlings, one concern is that if donor-derived oocytes are larger than the recipient’s original oocytes, they may not be able to pass through the recipient’s ovipositor. The oily bitterling was selected as the recipient in this study because its egg shape and size closely resemble those of the Tokyo bitterling, with both species exhibiting comparable egg widths of approximately 1.5–1.6 mm.

## Results

### Identification of optimal sampling stages for donor testes

**Fig. 1:**
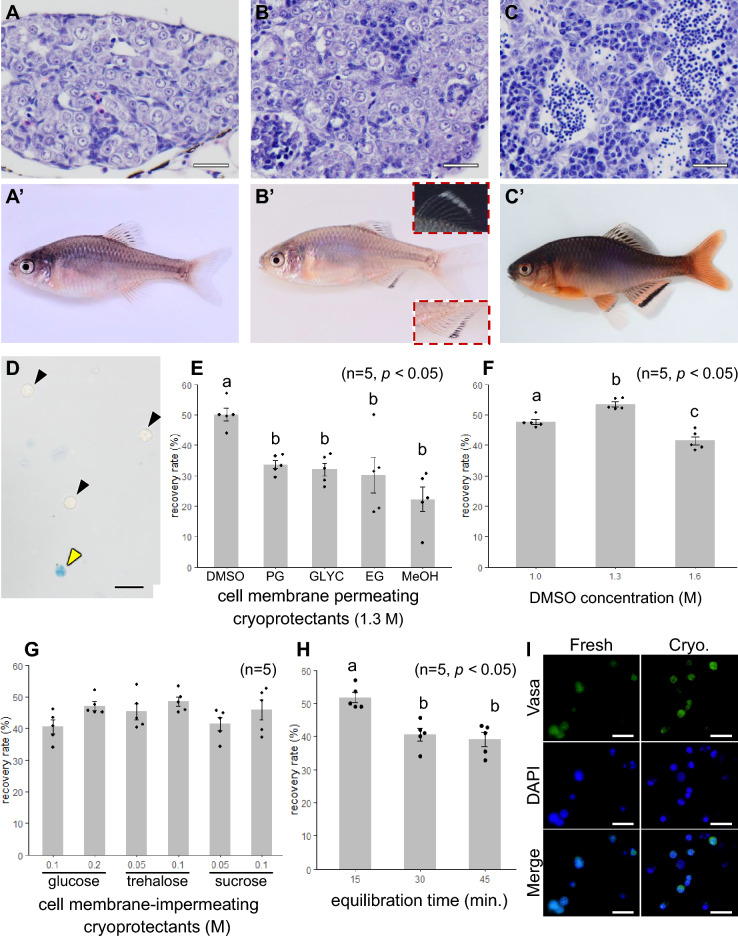
Optimization of cryopreservation conditions for Tokyo bitterling testes. A-C Haematoxylin‒eosin image of a fully immature (**A**) slightly mature (**B**) and mature testis (**C**) of Tokyo bitterling. A’-C’. The external morphology of Tokyo bitterling, which retains fully immature (A’), slightly mature (B’), and mature testes (C’). The black pattern on the outer edge of the anal fin and the white pattern on the outer tip of the dorsal fin (sign of nuptial colouration), which are indicators of donor fish selection, are shown in the insets. (**D**) Trypan blue-stained image of testicular cells showing live cells (black arrowhead) and dead cells (yellow arrowhead). (**E**) Recovery rates of testicular cells cryopreserved in a cryomedium containing 1.3 M DMSO, PG, GLYC, EG, and MeOH. (**F**) Recovery rates of testicular cells cryopreserved in cryomedium containing 1.0, 1.3, or 1.6 M DMSO. (**G**) Recovery rates of testicular cells cryopreserved in cryomedium containing 1.3 M DMSO and 0.1 or 0.2 M glucose or 0.05 or 0.1 M trehalose or sucrose. (**H**) Recovery rates of cryopreserved testicular cells after equilibration in a cryomedium containing 1.3 M DMSO and 0.1 M trehalose for 15, 30 and 45 min. (**I**) Fluorescence images of testicular cells after cryopreservation obtained by immunostaining with the Vasa antibody. DAPI staining was used to visualize the nucleus. Bars: 20 µm.

To facilitate efficient transplantation using testes from valuable individuals, we sought donor fish possessing large testes with a high abundance of type A spermatogonia (ASG), which are optimal for transplantation. Initially, sampling was based on the age in months, a parameter previously used for donor selection in various farmed species. However, testicular maturation in the Tokyo bitterling exhibited substantial individual variation, making age-based selection unreliable for identifying suitable donors. Therefore, the testes were evaluated based on their degree of maturation and classified into three categories: fully immature (Fig. 1A), slightly mature (Fig. 1B), and mature (Fig. 1C). The average body weights of the fish at the three maturation stages, corresponding to ages 4–6, 5–7, and 5–7 months, were 0.37 ± 0.04, 0.48 ± 0.07, and 0.79 ± 0.11 g, respectively (Table S1). First, fully immature Tokyo bitterlings presented a high frequency of type A spermatogonia (ASGs) (31.6 ± 2.4%; n = 8) (Fig. 1A, A’), but the absolute number of germ cells was extremely low, and the testes were very small (maximal diameter of the testis = 210.7 ± 18.3 µm; n = 8); thus, surgically isolating the testes was technically difficult and time-consuming. In contrast, mature individuals had large testes (maximal diameter of testis = 670.2 ± 72.9 µm; n = 6) but an extremely low frequency of ASGs (8.8 ± 1.4%; n = 6), making them unsuitable for use as donors (Fig. 1C, C’). Next, we selected males with slightly mature testes, intermediate between immature and mature. At this stage, Tokyo bitterling testes were large enough to be surgically removed (maximal diameter of testis = 420.5 ± 64.6 µm; n = 6), and ASGs comprised approximately 19.0 ± 2.1% (n = 6) of the total testicular cells (Fig. 1B), making them suitable for use as donors in transplantation experiments. We found that nuptial coloration can serve as a reliable indicator for determining the appropriate timing for donor sampling (Fig. 1A'-C'). In individuals at a slightly mature stage—considered suitable for use as donors—distinct features were observed, including black pigmentation along the outer margin of the anal fin and a white marking at the distal edge of the dorsal fin. These features appeared early during testicular development and coincided with the onset of nuptial coloration (Fig. 1B').

### Optimization of cryopreservation conditions for whole testes from Tokyo bitterling

Since a method to isolate only germ cells has not been established for Tokyo bitterling, we first optimized the recovery rate of whole testicular cells after trypan blue staining (Fig. 1D). The cell recovery rate of testicular cells cryopreserved in a cryomedium containing 1.3 M dimethyl sulfoxide (DMSO) was significantly greater than that of testicular cells cryopreserved in a cryomedium containing either 1.3 M propylene glycol (PG), glycerol (GLYC), ethylene glycol (EG), or methanol (MeOH) (Fig. 1E). The recovery rates of testicular cells cryopreserved in cryomedia containing different concentrations of DMSO (1.0, 1.3 or 1.6 M) were examined, and we found that cryomedium with 1.3 M DMSO resulted in significantly higher rates than those of the other concentrations (Fig. 1F). The recovery rate of testicular cells was then assessed when a cell membrane-impermeable cryoprotectant was added to the cryomedium containing 1.3 M DMSO. Among cryomedia supplemented with glucose (0.1 or 0.2 M), trehalose (0.05 or 0.1 M), or sucrose (0.05 or 0.1 M), 48.5 ± 1.5% of the cells were recovered with 0.1 M trehalose, representing the highest mean recovery rate observed (Fig. 1G), although the difference was not statistically significant compared to the other groups. Finally, the effect of the equilibration time of the cryomedia containing 1.3 M DMSO and 0.1 M trehalose on the testicular cells was assessed. The results revealed that the cell recovery rate was significantly greater when equilibration was performed for 15 min than when equilibration times of 30 and 45 min were used (Fig. 1H). In the next experiment, we used a Vasa antibody to confirm the presence of germ cells in these testicular cells cryopreserved with optimized conditions (1.3 M DMSO, 0.1 M trehalose, 15-min equilibration). As shown in Fig. 1I, more than half of the cryopreserved testicular cells were clearly positive for the Vasa antibody. Quantitative analysis revealed that the proportion of Vasa-positive cells (Vasa⁺/DAPI⁺) was 51.97 ± 3.61% before cryopreservation (30/52, 27/51, 24/53) and remained comparable at 50.98 ± 2.66% after cryopreservation (28/50, 25/50, 23/49), showing no statistically significant difference between the two groups.

### Transplantation of cryopreserved testicular cells

**Fig. 2:**
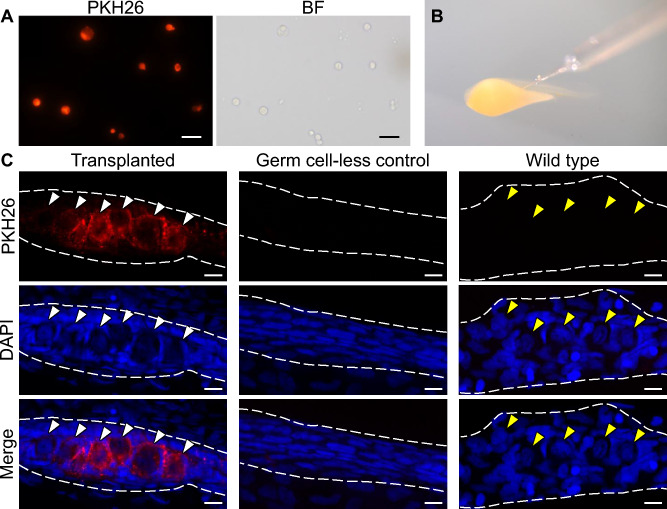
Intraperitoneal transplantation of Tokyo bitterling testicular cells into oily bitterling larvae. (**A**) Fluorescence image of Tokyo bitterling testicular cells isolated and fluorescently labelled with PKH26. BF shows a bright field view. Bars: 20 µm. (**B**) Intraperitoneal transplantation of testicular cells into oily bitterling larvae. (**C**) Fluorescence images of the immature gonads of oily bitterling recipients at 18 days after transplantation. Transplanted, germ cell-less control, and wild-type represent the gonads of *dnd*-KD recipients receiving donor germ cells, those of *dnd*-KD recipients without transplantation, and those of wild-type controls, respectively. The white arrowheads indicate donor-derived germ cells labelled with PKH26. The yellow arrowheads indicate endogenous germ cells with round-shaped nuclei. Bars: 10 µm.

The donor testes were enzymatically dissociated, and the resulting testicular cells were fluorescently labelled with PKH26 (Fig. 2A). Approximately 10,000 donor testicular cells fluorescently labelled with PKH26 were microinjected into the abdominal cavity at 4 days post-fertilization (dpf) into oily bitterling larvae lacking endogenous germ cells due to *dnd* knockdown (Fig. 2B). The survival rate of the recipients 18 days after transplantation was 87.6 ± 3.8%, which was lower than that of the control (100%) but high enough for practical use. In nearly 40% (4/10, 4/10, 3/10) of the recipient genital ridges, PKH26-positive cells with large, oval, weakly DAPI-stained nuclei were observed (Fig. 2C). No red fluorescent cells were observed in the *dnd*-knockdown control (germ cell-less control) or wild-type control genital ridges (Fig. 2C). In the former, cells possessing nuclei with germ-cell characteristics were not observed.

### Production of functional gametes derived from cryopreserved germ cells

**Fig. 3:**
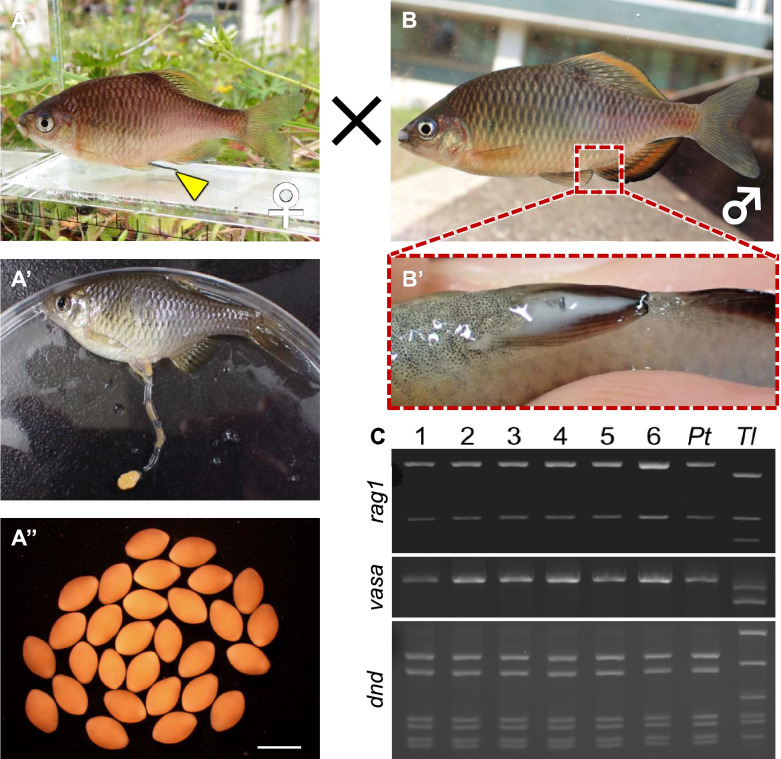
Production of eggs and sperm derived from donor Tokyo bitterling by oily bitterling recipients. (**A**) Mature female recipient possessing donor germ cells. The ovipositor is indicated by a yellow arrowhead. A’. Eggs obtained by the squeeze method were passed through the ovipositor. A^”^. Eggs obtained from A. Bar: 2 mm. (**B**) Male recipient possessing donor germ cells. B^’^. Milt obtained by pressure on the abdomen of B. (**C**) PCR analysis of DNA extracted from milt produced by recipients #1–6 with the *rag1*, *vasa* and *dnd* primers. Amplicons for the *rag1* and *dnd* genes were digested with *Sac*I and *Mbo*I, respectively. Tokyo bitterling (*Pt*) and oily bitterling (*Tl*) sperm DNA were also analysed as controls.

**Fig. 4:**
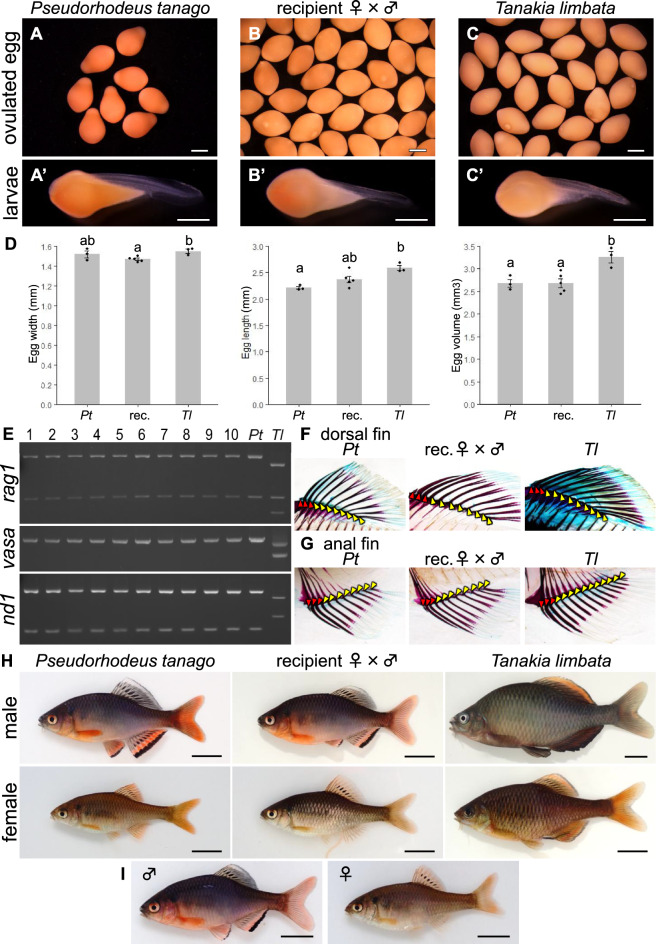
Production of donor-derived Tokyo bitterling offspring by *dnd*-KD oily bitterling recipients. (**A-C**) Ovulated eggs produced by Tokyo bitterling (*Pseudorhodeus tanago*), recipient, and oily bitterling (*Tanakia limbata*) females. A^’^-C^’^. Larvae hatched from A-C eggs fertilized with sperm produced by Tokyo bitterling, recipient, and oily bitterling males. Bars: 1 mm. (**D**) Width, length and volume of ovulated eggs produced by Tokyo bitterling (*Pt*), recipient (rec.) and oily bitterling (*Tl*). Significant differences among the groups are indicated by *lowercase letters* (*p* < 0.05). (**E**) PCR (-RFLP) analysis of DNA extracted from the resulting offspring (#1–10) with the *rag1*, *vasa* and *nd1* primers. Amplicons for the *rag1* and *dnd* genes were digested with *Sac*I and *Mbo*I, respectively. Tokyo bitterling (*Pt*) and oily bitterling (*Tl*) larval DNA samples were also analysed as controls. (**F, G**) Dorsal (F) and anal (G) fins of clearing and staining samples of Tokyo bitterling (*Pt*), donor-derived offspring (rec.♀ × ♂), and oily bitterling (*Tl*). Red and yellow arrowheads indicate unbranched and branched fin rays, respectively. (**H**) External view of adult-stage Tokyo bitterling (*P. tanago*), offspring produced by recipient parents, and oily bitterling (*T. limbata*). Bars: 1 cm. (**I**) F2 offspring produced from donor-derived offspring.

Six and a half months after transplantation, 11 (males = 6; females = 5) of the 16 surviving recipients matured and produced gametes (Fig. 3A, B). The five females had long ovipositors (Fig. 3A and A’), and the six males presented distinct nuptial colouration (Fig. 3B). First, genetic analysis was performed to determine whether the sperm produced by the male recipient was derived from cryopreserved donor cells. We found that all the male recipients produced only donor-derived sperm showing identical DNA banding patterns to those of the donor Tokyo bitterling (Fig. 3C). Ovulated eggs were then squeezed from the female recipients (Fig. 3A', A''), and their shape, egg number, size and volume were analysed and compared with those of Tokyo bitterling and oily bitterling. We found no notable shape differences between any of the ovulated eggs (Fig. 4A-C). We obtained 36.5 ± 2.9 eggs from the recipients, 7.1 ± 0.9 eggs from the Tokyo bitterlings, and 34.6 ± 3.6 eggs from the control oily bitterlings, with only the number of Tokyo bitterling eggs being significantly lower (Table 1). The widths of eggs from Tokyo bitterlings, recipients, and oily bitterlings were 1.52 ± 0.03 mm, 1.47 ± 0.01 mm, and 1.55 ± 0.02 mm, respectively, with significantly larger widths for oily bitterling eggs than for recipient-produced eggs (Fig. 4D). The lengths of eggs produced by Tokyo bitterlings, recipients, and oily bitterlings were 2.21 ± 0.03 mm, 2.37 ± 0.06 mm, and 2.58 ± 0.05 mm, respectively, with oily bitterling eggs being significantly longer than those of Tokyo bitterlings (Fig. 4D). The egg volumes of Tokyo bitterlings, recipients, and oily bitterlings were 2.68 ± 0.09 mm^3^, 2.68 ± 0.10 mm^3^, and 3.26 ± 0.13 mm^3^, respectively, with oily bitterling eggs being significantly larger in volume than those produced by Tokyo bitterlings or their recipients (Fig. 4D). For confirmation that the resulting gametes were functional, the eggs were then fertilized with sperm from the recipients, and the morphology and hatching rate of the hatchlings were compared with those produced by Tokyo bitterling and oily bitterling. We found no notable morphological differences between any of the larvae (Fig. 4A'-C'). The hatchability of the next generation produced by the fertilization of eggs and sperm from the recipients was 82.5 ± 3.1%, that of the Tokyo bitterling was 95.8 ± 2.4%, and that of the oily bitterling was 88.6 ± 1.2%, with no significant differences among these values. (Table 1). Genetic analysis of the resulting larvae produced by the recipient males and females revealed that all the larvae carried Tokyo bitterling genotypes, and no sign of contaminating oily bitterling DNA was detected (Fig. 4E). We then measured the number of fin rays, a morphological feature that distinguishes Tokyo bitterling from oily bitterling. The dorsal fins were ⅲ + 8 for Tokyo bitterling, ⅲ + 8 for the resulting offspring produced by the recipients, and ⅲ + 9 for oily bitterling, and the anal fins were ⅲ + 8 for Tokyo bitterling, ⅲ + 8 for the resulting offspring produced by the recipients, and ⅲ + 10 or 11 for oily bitterling (n = 3, Fig. 4F, G). When the external morphology of each fish was observed at maturity, the nuptial colour of the resulting offspring produced by the recipients, the white pattern on the outer tip of the dorsal fin, and the black and orange patterns on the outer edge of the ventral and anal fins were the same as those of Tokyo bitterling (Fig. 4H). In the progeny derived from mating male and female recipients, the observed sex ratio was 230 males to 74 females, which was close to the theoretical ratio of 3:1. The F1 generation produced by the recipients matured normally and produced eggs and sperm. The hatching rate of the F2 generation was 88.9 ± 11.1% (n = 3; Table S2), and they continued to develop normally (Fig. 4I).

**Table 1:** Number of Eggs and Hatched Larvae in Recipients, Tokyo Bitterling, and Oily Bitterling.

Parent		No. of eggs	No. of hatching larvae	Hatching rate (%)
Male	Female			
Recipient	Recipient	36.5 ± 2.9	30.9 ± 1.9	82.5 ± 3.1
Tokyo Bit	Tokyo Bit	7.1 ± 0.9	6.9 ± 0.8	95.8 ± 2.4
Oily Bit	Oily Bit	34.6 ± 3.6	30.6 ± 2.8	88.6 ± 1.2

## Discussion

In this study, we succeeded in producing functional Tokyo bitterling gametes in sterile oily bitterling recipients transplanted with cryopreserved Tokyo bitterling testicular cells. The recovery rate of testicular cells after thawing was as high as approximately 50%, indicating that the cryopreservation method we developed for Tokyo bitterling germ cells in this study is sufficient for practical use. The number of donor-derived Tokyo bitterling eggs produced by the recipients was equal to that produced by the recipient oily bitterlings, reaching approximately five times that produced by Tokyo bitterlings. Next-generation individuals produced by the fertilization of frozen cell-derived eggs and sperm developed normally, and after normal maturation, normal F2 individuals were also successfully obtained. These results indicate that the combination of cryopreservation and transplantation of testicular germ cells can be a powerful tool for conserving the Tokyo bitterling on the brink of extinction.

Sperm cryopreservation has been performed in fish as a method of preserving genetic resources; however, because of the difficulty of cryopreserving eggs and embryos^19–22^, it is not possible to restore individuals using only cryopreserved materials. Researchers have proposed that it may be possible to restore live individuals by combining cryopreserved sperm and androgenesis using eggs of other species^31^. Androgenesis has already been induced in many fish species, including salmonids and cyprinids^32^. In addition, Babiak et al*.* (2002) and Bercsényi et al*.* (1998) successfully demonstrated androgenesis via cryopreserved rainbow trout sperm and interspecific androgenesis by using carp eggs with sperm from goldfish^33,34^. These studies suggested that even if an endangered species becomes extinct, individuals could be restored if only the cryopreserved sperm of the target species and eggs of closely related species were available. However, even the best-studied species, such as carp and rainbow trout, present very low initial survival rates^32^. In addition, because androgenesis induces chromosome duplication, the nuclear genome becomes invariably homozygous, and concerns have been raised about the expression of detrimental recessive genes. More importantly, mitochondrial DNA (mtDNA) is maternally inherited, so even if sperm cryopreservation is possible, the mtDNA of the resulting offspring will be of egg origin. In other words, the offspring become nuclear‒cytoplasmic hybrids possessing nuclear DNA derived from the sperm and mtDNA derived from the eggs. Indeed, a study by Bercsényi et al*.* (1998) reported that androgenic offspring carried the nuclear genotype of goldfish and the mitochondrial genotype of carp^34^. Given the above, it is not possible in principle to combine sperm cryopreservation and androgenesis to preserve the complete genome of an endangered species. In the surrogate broodstock technique, in contrast, both the nuclear genome and mtDNA that are passed on to the next generation are derived from donor cells. In fact, in this study, the genotypes of the mtDNA of the next generation produced by the recipient oily bitterling were identical to those produced by the donor Tokyo bitterling.

The surrogate broodstock technique can also contribute to the maintenance of the genetic diversity of target species. Sato et al*.* (2014) transplanted germ cells derived from three donor strains of rainbow trout into a recipient and successfully produced eggs and sperm of all the donor strains from the recipient^35^. This method also makes it possible to produce genetically diverse gametes from a small number of individual recipients. Therefore, the technology established in this study may overcome the weaknesses of sperm cryopreservation and androgenesis.

This study is the first report of the production of both eggs and sperm from germ cells cryopreserved in liquid nitrogen using fish that have actually been driven to the brink of extinction, with other fish species used as surrogate broodstock. This report is a very important step towards ex situ conservation of many endangered species in the future. In general, it has been difficult to produce offspring via intergenus germ cell transplantation^24^. Although there are several examples of surrogate-produced sperm derived from donors belonging to different genera, successful production of functional eggs is limited to experiments with slender bittering and Chinese rosy bitterling^24,36^, carp and goldfish^37,38^, and the present study. Thus, functional egg production in intergenus transplants, including those in this study, is limited to experiments with *Cyprinidae*. Although the reasons for the success of intergenus transplantation only in *Cyprinidae* are not clear at this time, elucidation of the causes of these successes will increase the success rate of germ cell transplantation between distantly related species in the future.

When the germ cells of Tokyo bitterling, which spawn small numbers of eggs per spawn (1–14 eggs/spawning)^8,10^, are transplanted into oily bitterling, which spawn more eggs per spawn, the oily bitterling recipients produce approximately 35 Tokyo bitterling eggs as the control oily bitterling. Thus, by using surrogate broodstock in this study, we succeeded in obtaining an average of approximately five times more eggs of Tokyo bitterling in a single spawning than that of control Tokyo bitterling. The results of this study indicate that the selection of recipients with high fecundity is a major advantage in the conservation of endangered species. The reason why the oily bitterling is able to produce such a large number of eggs remains unclear; however, its average body weight is approximately five times greater than that of the Tokyo bitterling. These findings suggest that the selection of a larger recipient species is a critical factor in increasing the number of donor-derived eggs. As shown by the above results, the number of gametes is dependent on the recipient species rather than the donor species. Previous reports using experimental fish species, such as *Danio*^39^ and carp^38^, also supported this result.

In this study, the volume of the eggs produced by the recipient was similar to that of the donor, but distinct from that of the recipient species itself. A similar result has also been observed in germ cell transplantation experiments between the Chinese rosy bitterling (*Rhodeus ocellatus ocellatus*) and the slender bitterling (*Tanakia lanceolata*)^36^. Furthermore, in other groups of fish, it has been demonstrated that the donor species exerts a strong influence on the egg size produced by the recipient^37,40,41^, suggesting that this tendency is a phenomenon conserved across fishes.

The original habitat of Tokyo bitterling was floodplains^7^. However, the habitat of Tokyo bitterling changed drastically approximately 2,000 years ago, when humans began rice farming. Rice farming has created many tiny ditches between rice fields, creating a new habitat for Tokyo bitterling^5^. However, much of the floodplain was lost during the past century because of river channel modifications and the construction of concrete revetments. As a result, Tokyo bitterling habitat became restricted to artificial small ditches and reservoirs. Furthermore, since the 1960 s, the banks of many tiny ditches between rice fields have been concreted, and weirs have been installed. As a result, most of the 45 habitats known for Tokyo bitterling by around the year 2000 have been lost to date, and the size of the remaining populations has decreased significantly over the years^42^. Saitoh et al*.* (2017) reported that haplotypes in the mitochondria of Tokyo bitterling from many of these populations are monotypic and extremely inbred^42^. Furthermore, Kubota et al*.* (2010) conducted microsatellite analyses in four wild populations of Tokyo bitterling and reported that all of them have small effective population sizes and have experienced or are experiencing serious bottlenecks^43^. These findings indicate that relying solely on habitat restoration may not be sufficient to ensure the stable persistence of Tokyo bitterling populations. The method developed in this study enables the semipermanent preservation of the genetic resources of wild Tokyo bitterling without any genetic alterations. In other words, this method allows the long-term storage of genetic material and the production of healthy next-generation individuals as needed until a suitable habitat for Tokyo bitterling is restored. While captive breeding carries the risk of genetic alterations and inbreeding over successive generations, it is noteworthy that cryopreserving germ cells using this method eliminates the risk of genetic alteration, regardless of the storage duration. The methodology developed in this study could be applied to other bitterling species soon and potentially to other endangered species as well.

## Materials and methods

### Fish

All experimental procedures were followed in accordance with protocols approved by Institutional Animal Care and Use Committee (IACUC) of Tokyo University of Marine Science and Technology (TUMSAT), and all methods are reported in accordance with ARRIVE guidelines. All experiments were performed in accordance with the relevant guidelines. Donor Tokyo bitterlings were provided by the Tochigi Prefectural Fisheries Experiment Station, and oily bitterlings were purchased from an ornamental fish store in Saitama, Japan. All experimental fish were reared based on a protocol with some modifications from Octavera and Yoshizaki (2019)^44^. Specifically, all experimental fish were kept in 45- or 60-L tanks with 14 h of light, a 10-h dark cycle and a water temperature of 20 ± 1 °C. All the experimental fish were fed brine shrimp (*Artemia* nauplii) twice daily and a commercial formula diet (Otohime B2; Marubeni Nisshin Feed Co., Ltd., Japan) once daily. Larvae were produced by artificial insemination, and the fertilized eggs up to 25 dpf were reared in an incubator at 20 °C. From 25–35 dpf, when yolk absorption was complete, brine shrimp were fed once a day with 14 h of daylight, a 10-h dark cycle and a 20 °C water temperature in 3 L tanks with gentle aeration. The collection of eggs and sperm from the fish was performed after anesthetizing them by immersion in a 2-phenoxyethanol solution at a concentration of 250 ppm. Fish were monitored regularly for signs of distress or abnormal behavior during the experimental procedures. Humane endpoints were defined as loss of equilibrium, or failure to respond to external stimuli. In such cases, the affected fish were euthanized immediately using an overdose of anesthetic (e.g., 2-phenoxyethanol at 400 ppm).

### Recipient preparation

On the basis of Octavera and Yoshizaki (2019), in vitro artificial insemination was performed by applying gentle pressure to the abdomens of oily bitterling males whose nuptial colour was fully expressed and oily bitterling females whose ovipositor was elongated. Modified in part from Octavera and Yoshizaki (2019)^44^, 3 ng of morpholino antisense oligonucleotide (5'- AACCTGATGCTGTCCCTCCATGT-3') against the *dnd* gene was injected into fertilized eggs to produce sterile recipients lacking endogenous germ cells. 4-dpf hatchlings were used as recipients.

### Optimization of the maturation stages of the donors used for germ cell transplantation

For determination of the suitable maturational stages of donor testes used for germ cell transplantation, the testes at various maturational stages were analysed by morphological and histological methods. Surgically removed testes were stored on ice in Leibovitz’s L-15 medium (L-15; Gibco, USA) (pH = 7.8) containing 10% (vol/vol) FBS (Gibco) until they were used in the following various experiments. For histological studies, the isolated testes were fixed in Bouin’s fixative for 18 h at 4 °C. Fixed tissues were dehydrated in a graded ethanol series and embedded in paraffin. Transverse sections of 4 µm thickness were prepared at a total of five locations, one every 20 µm near the centre of the testis, which was expected to have the largest diameter. The resulting sections were stained with haematoxylin and eosin. The testis sections from each maturational stage were observed under an optical microscope (BX53; Olympus Corporation, Japan), and the maximum diameter of the testis was measured using a CellSens Standard (Olympus Corporation). The transverse sections of the testes were then photographed using a CCD camera (DP74; Olympus Corporation). A 25 mm grid line was drawn across the histology images (96 dpi, 1600 × 1200 px), and the number of cells and ASGs present at the intersections were counted. The frequency of ASGs was calculated according to the following formula: frequency of ASGs (%) = number of ASGs at intersections/number of testicular cells at all intersections × 100. In this study, germ cells that were round in shape with a diameter of ≥ 7 µm were counted as ASGs.

### Optimization of cryopreservation conditions for whole testes

The isolated testes were also used to optimize the cryopreservation conditions. To determine the number of surviving cells after freezing and thawing relative to the number of testis cells before freezing, we optimized the freezing conditions of the testes. Because of the small size difference between the left and right testes of Tokyo bitterlings, the cell recovery rate was calculated by subjecting the left side to cell dispersion immediately after removal and the right side to cell dispersion after freezing and thawing. The right-sided testes were frozen by the slow freezing method as follows. The excised testes were transferred to 1.2 mL cryotubes (TPP Techno Plastic Products AG, Switzerland). The tubes were filled with 500 µL of L-15, which contained membrane-permeating cryoprotectants (DMSO, PG, GLYC, EG, or MeOH) [all from Sigma‒Aldrich (USA), 1.0 M, 1.3 M or 1.6 M], membrane-nonpermeating cryoprotectants [0.1 M or 0.2 M D-(+)-glucose (Sigma‒Aldrich), 0.05 M or 0.1 M D-(+)-trehalose dihydrate (Sigma‒Aldrich), 0.05 M or 0.1 M sucrose (FUJIFILM Wako Pure Chemical Corporation, Japan)], 1.5% (wt/vol) BSA (Sigma‒Aldrich), 1 mM CaCl_2_−2H_2_O (FUJIFILM Wako Pure Chemical Corporation), and 0.5 mM MgCl_2_−6H_2_O (FUJIFILM Wako Pure Chemical Corporation). The testes were equilibrated with cryobuffer on ice for 15 min, stored in a Bicell plastic freezing container (Nihon Freezer Co., Ltd., Japan) and placed in a deep freezer (CLN-35C, Nihon Freezer Co., Ltd.) for 90 min to cool to −80 °C at a rate of −1 °C/min. The samples were then stored in liquid nitrogen. After freezing for at least 24 h, the cryotubes containing testes were thawed by shaking in a water bath at 20 °C for approximately 1 min. Following thawing, the testes were transferred to twelve-well plates, and the cryomedium was exchanged three times with 4 mL of L-15 medium supplemented with 10% FBS to eliminate residual cryoprotectants. When the testes were used for transplantation, whole testes from 10 fish were used and frozen in each tube.

### Assessment of cell viability

For evaluation of the cell recovery rates before and after freezing, the protocol of Lee et al*.* (2013) was partially modified to include 0.51 U/mL collagenase H (Roche, Switzerland), 500 U/mL dispase II (FUJIFILM Wako Pure Chemical Corporation), and 450 U/mL DNase I (Sigma‒Aldrich) in a total of 500 µL of L-15 medium to dissociate each of the fresh and cryopreserved testes. The resulting testicular cells were passed through a 42 µm pore diameter nylon mesh (NBC Meshtec, Inc., Japan) to remove undissociated cell clumps. Cell viability was assessed using the trypan blue exclusion method^45^. Specifically, blue-stained cells were considered dead cells, and unstained cells were considered live cells. The cell recovery rate was calculated according to the following formula: cell recovery rate (%) = (number of viable cryopreserved testicular cells/weight of cryopreserved testis)/(number of fresh testicular cells/weight of fresh testis) × 100. All the cells, excluding sperm, were counted using a hemocytometer, and each dataset consisted of three independent trials per experiment.

For confirmation of the presence of sufficient germ cells after freezing, a portion of the testicular cell suspension was subjected to immunocytochemistry with a Vasa antibody against a cell smear, following the methodology described by Kise et al*.* (2012)^46^, with some modifications. Approximately 10,000 cells were fixed using Tissue-Tek Ufix (Sakura Finetek Japan, Japan) for 5 min. Smears were then prepared on adhesive silane-coated glass slides (MAS-GP; Matsunami Glass Ind., Ltd., Japan). After drying overnight at 42 °C, the slides were rinsed with PBS (-). Immunofluorescence staining was used to determine the presence of germ cells. An anti-Vasa antibody (1:5000; Abcam 209,710; Abcam, UK) was used as the primary antibody, and a secondary antibody (1:200; Alexa Fluor 488-conjugated goat anti-rabbit IgG; A-11008; Invitrogen, USA) was applied to the smears. 4',6-Diamidino-2-phenylindole, dihydrochloride (DAPI) (Thermo Fisher Scientific, USA) was prepared to 14.3 µM by dilution with PBS(-) and was applied to the smears before observation. Fluorescence signals were obtained using a fluorescence microscope (BX53; Olympus Corporation) equipped with appropriate filters (U-MNIBA3 for Alexa 488; U-MNUA2 for DAPI; both Olympus Corporation).

### Spermatogonial transplantation

To observe the behaviour of Tokyo bitterling testicular cells in the recipient abdominal cavity after transplantation, we labelled frozen and thawed testicular cells with PKH26 (Sigma‒Aldrich) following the protocol of Takeuchi et al*.* (2009)^47^. To prevent cell death and increase cell viability, we added Y-27632 dihydrochloride (Sigma‒Aldrich) to the fluorescently labelled testicular cell suspension to a concentration of 50 µM; the sample was subsequently incubated at 4 °C for 16 h. To remove cell clumps once more, we passed the testicular cell suspension through a nylon mesh (NBC Meshtec, Inc.) with a pore size of 20 µm immediately before transplantation.

The transplantation of Tokyo bitterling testicular cells into the oily bitterling larvae was performed using a micromanipulator (MP-2R; Narishige Scientific Instruments Laboratory, Japan) and a microinjector (IM-9A; Narishige Scientific Instruments Laboratory) mounted on a stereomicroscope (SZX10; Olympus Corporation). Approximately 10,000 cells fluorescently labelled with PKH26 were transplanted into the abdominal cavity of sterilized oily bitterling larvae at 4 dpf (Fig. 2C). The recipients were allowed to recover for 2 days in a recovery tank containing 0.9% NaCl dissolved in dechlorinated 20 °C tap water and then reared in dechlorinated 20 °C tap water. At 18 days after transplantation, the recipients were anaesthetized and immersed in Tissue-Tek Ufix for 10 min for fixation. The body cavity was opened, and the samples were immersed in 14.3 µM DAPI solution prepared with PBS(-) for 10 min. The gonads were then surgically removed, placed on glass slides (MAS-GP) with an appropriate amount of PBS(-) dripped, and sealed with a micro cover glass (Muto Pure Chemicals Co., Ltd., Japan). With an all-in-one fluorescence microscope (BZ-X810; KEYENCE Corporation, Japan) equipped with a BZ-X filter TRITC (KEYENCE Corporation) and a BZ-X filter DAPI-V (KEYENCE Corporation), donor cells fluorescently labelled with PKH26 were observed to confirm donor-derived cell incorporation into the recipient gonadal anlagen, and the shape of their nuclei was fluorescently observed.

### Progeny test

The resulting recipients were reared to maturity. Semen was collected from six 6.5-month-old recipient males with nuptial colouration, and sperm DNA was extracted via a DNA Extraction Kit (QIAGEN, Netherlands). PCR was performed via the use of 10 ng of extracted DNA as a template with primers against *recombination-activating gene 1* (*rag1*), *vasa*, and the *dead-end gene* (*dnd*) (Table S3). PCR for *rag1* and *vasa* was performed using *Ex Taq* polymerase (TaKaRa Bio, Inc., Japan) under the following conditions: one cycle of 94 °C for 3 min; 30 cycles of 94 °C for 30 s, 65 °C or 64 °C for 30 s (*rag*1 or *vasa*); 72 °C for 1 min 15 s or 2 min 30 s (*rag*1 or *vasa*); and a final elongation step at 72 °C for 3 min. PCR with *dnd* primers was performed using PRIME STAR Max DNA Polymerase (TaKaRa Bio, Inc.) under the following conditions: one cycle at 98 °C for 30 s; 30 cycles at 98 °C for 10 s, 55 °C for 5 s, and 72 °C for 15 s. The *dnd* PCR products were then subjected to nested PCR using the primers in Table S3 (dnd_F2, dnd_R2) under the following conditions: one cycle of 94 °C for 3 min; 30 cycles of 94 °C for 30 s, 63 °C for 30 s, and 72 °C for 2 min 30 s; and a final elongation step at 72 °C for 3 min. *Sac*I (TaKaRa Bio, Inc.) and *Mbo*I (TaKaRa Bio, Inc.) were used to detect polymorphisms of *rag 1* and *dnd*, respectively. The cleaved fragments were electrophoresed on 2.0% agarose gels (Nippon Gene Co., Ltd., Japan). The *vasa* PCR product was electrophoresed on 0.7% agarose gels (Nippon Gene Co., Ltd.) to detect the size differences of the amplicons.

Eggs obtained from five 6.5-month-old recipient females with elongated ovipositors were fertilized with sperm produced by male recipients. DNA was extracted from the hatchlings to identify their genetic origin. With 10 ng of extracted DNA as a template, species identification was performed using *rag1* and *vasa* as described above. PCR was also performed using primers specific for *NADH dehydrogenase subunit 1* (*nd1*) to identify the origin of the mitochondrial DNA (Table S3) *Ex Taq* Polymerase (TaKaRa Bio, Inc.) was used under the following conditions: one cycle at 94 °C for 3 min; 30 cycles at 94 °C for 30 s, 60 °C for 30 s, and 72 °C for 30 s; and a final elongation step at 72 °C for 3 min. The PCR products were subjected to restriction enzyme treatment with *Ssp*I (TaKaRa Bio, Inc.) at 37 °C for 3 h. The *nd1* cleavage fragments were electrophoresed on 2.0% agarose gels (Nippon Gene Co., Ltd.).

Cleared and stained skeletal samples of Tokyo bitterling, oily bitterling, and offspring produced by the recipients were prepared by the method modified from Potthoff et al*.* (1984)^48^. The fish were fixed in 10% formalin for 1‒2 weeks, immersed in 50% and 100% ethanol for 1 day each, and dehydrated. The samples were placed in a staining solution of Alcian blue 8GX (Sigma‒Aldrich) diluted in a mixture of 70% ethanol and 30% glacial acetic acid at 0.2 mg/mL, and the cartilage was stained at 4 °C for up to 24 h. The samples were then transferred to a saturated sodium tetraborate solution (Kokusan Chemical Co., Ltd., Japan) to neutralize the acid in the samples. The samples were then immersed in a 30% sodium tetraborate solution containing 10 ng/mL trypsin (FUJIFILM Wako Pure Chemical Corporation) at 37 °C for several weeks for clarification. Once the samples were somewhat clear, they were immersed in 0.5% potassium hydroxide solution (Kokusan Chemical Co., Ltd.) containing 0.2% Alizarin Red S (FUJIFILM Wako Pure Chemical Corporation) for up to 3 days to stain hard bone. The samples were then immersed in glycerin-0.5% potassium hydroxide solution (10, 20, 30, 40, 50, and 60% glycerin) for 1 day each, glycerin-0.5% potassium hydroxide solution (70, 80, and 90% glycerin) for several days each, and finally preserved in 100% glycerin. The number of fin rays was counted under a stereomicroscope (SZX10; Olympus Corporation).

### Egg shape, size, and volume analyses

The shape, size and volume of the eggs produced by the recipient were measured according to the methods of Octavera et al. (2023)^36^. These values were obtained for all eggs in each group.

### Statistical analysis

The data are presented as the means ± SEMs. Statistical significance was determined by one-way ANOVA followed by the Tukey or Tukey‒Kramer test. For all the statistical tests, differences were considered statistically significant when *p* < 0.05.

## Supplementary Information

Supplementary Information 1.

Supplementary Information 2.

Supplementary Information 3.

Supplementary Information 4.

## Data Availability

The data supporting the results of this study are available within the paper and supplementary information. All unique materials used are available from the authors on reasonable request. Source data are provided with this paper.

## References

[1] Strayer, D. L. & Dudgeon, D. Freshwater biodiversity conservation: recent progress and future challenges. *J. North Am. Benthol. Soc.* **29**, 344–358 (2010).

[2] Díaz, S. *et al.* Summary for policymakers of the global assessment report on biodiversity and ecosystem services of the Intergovernmental Science-Policy Platform on Biodiversity and Ecosystem Services (IPBES, 2019)

[3] Sayer, C. A. et al. One-quarter of freshwater fauna threatened with extinction. *Nature* **638**, 138–145 (2025). 39779863 10.1038/s41586-024-08375-z PMC11798842

[4] Smith, C., Reichard, M., Jurajda, P. & Przybylski, M. The reproductive ecology of the European bitterling (*Rhodeus sericeus*). *J. Zool.* **262**, 107–124 (2004).

[5] Mochizuki, K. *Tanakia tanago.* In “Circumstances in Endangered Japanese Freshwater Fishes and Their Production” (ed. Nagata, Y. & Hosoya, K.) 64–75 (Midori-shobo, 1997).

[6] Arai, R. *Tanakia tanago.* In “Threatened wildlife of Japan-Red Data Book, 2^nd^ edition” (ed. Ministry of Environment.) (Japan Wildlife Research Center, 2003).

[7] Kitamura, J. & Uchiyama, R. Bitterling fishes of Japan (Yama-kei Publishers, 2020).

[8] Nakamura, M. Cyprinid Fishes of Japan 83–89 (Research Institute for Natural Resources, 1969).

[9] Taki, Y. *Tanakia tanago.* In “Basic Data of Rare Wild Aquatic Organisms in Japan” (ed. Fisheries Agency in Japan (Ⅰ)) 364–371 (Fisheries Resource Conservation Agency in Japan, 1994).

[10] Inugi, Y., Shiraishi, Y., Iizima, K. & Akiyama, N. Mechanism of *Tanakia tanago* spawning associated with the branchial cavity of a bivalve. *Aquacult. Sci.* **70**, 75–84 (2022).

[11] Tsunagawa, T., Sakai, T., Yoshida, Y., Kubota, H. & Sagawa, S. An approach to conservation of the endangered Tokyo bitterling, *Tanakia tanago*, in a natural habitat in southeastern Tochigi - Habitat requirement assessment and habitat restoration for juveniles. *Ecol. Civil Eng.* **15**, 249–255 (2012).

[12] Kohara, S., Murai, R., Yoshida, Y. & Kobori, I. Aquatic habitat survey including rare fishes -Survey of Tokyo bitterling habitat conditions-. *Bulletin of the Tochigi Prefectural Fisheries Experiment Station* **67**, 50–52 (2023).

[13] Suguro, N. An Endangered Freshwater Fishes of Kanagawa Prefecture-Ⅳ. *Kanagawa Prefectural Fisheries Experimental Station* **10**, 13–28 (2019).

[14] Hartl, D. L. & Clark, A. G. Principles of population genetics, 3^rd^ edition (Sinauer Associates Inc., 1997).

[15] Saccheri, I. et al. Inbreeding and extinction in a butterfly metapopulation. *Nature* **392**, 491–494 (1998).

[16] Frankham, R., Briscoe, D. A. & Ballou, J. D. Introduction to conservation genetics. (Cambridge University Press, 2002).

[17] Spielman, D., Brook, B. W. & Frankham, R. Most species are not driven to extinction before genetic factors impact them. *Proc. Natl. Acad. Sci. U. S. A.* **101**, 15261–15264 (2004). 15477597 10.1073/pnas.0403809101 PMC524053

[18] Cabrita, E. et al. Cryopreservation of fish sperm: applications and perspectives. *J. Appl. Ichthyol.* **26**, 623–635 (2010).

[19] Zhang, T. & Rawson, D. M. Studies on Chilling Sensitivity of Zebrafish (*Brachydanio rerio*) Embryos. *Cryobiology* **32**, 239–246 (1995).

[20] Hagedorn, M., Kleinhans, F. W., Artemov, D. & Pilatus, U. Characterization of a major permeability barrier in the zebrafish embryo. *Biol. Reprod.* **59**, 1240–1250 (1998). 9780333 10.1095/biolreprod59.5.1240

[21] Chao, N.-H. & Liao, I. C. Cryopreservation of finfish and shellfish gametes and embryos. *Aquaculture* **197**, 161–189 (2001).

[22] Diwan, A. D. et al. Cryobanking of fish and shellfish egg, embryos and larvae an overview. *Front. Mar. Sci.*10.3389/fmars.2020.00251 (2020).

[23] Yoshizaki, G. & Lee, S. Production of live fish derived from frozen germ cells via germ cell transplantation. *Stem Cell Res.* **29**, 103–110 (2018). 29649725 10.1016/j.scr.2018.03.015

[24] Yoshizaki, G. & Yazawa, R. Application of surrogate broodstock technology in aquaculture. *Fish. Sci.* **85**, 429–437 (2019).

[25] Okutsu, T., Suzuki, K., Takeuchi, Y., Takeuchi, T. & Yoshizaki, G. Testicular germ cells can colonize sexually undifferentiated embryonic gonad and produce functional eggs in fish. *Proc. Natl. Acad. Sci. U.S.A.* **103**, 2725–2729 (2006). 16473947 10.1073/pnas.0509218103 PMC1413788

[26] Okutsu, T., Shikina, S., Kanno, M., Takeuchi, Y. & Yoshizaki, G. Production of trout offspring from triploid salmon parents. *Science* **317**, 1517 (2007). 17872437 10.1126/science.1145626

[27] Yoshizaki, G. et al. Sexual plasticity of ovarian germ cells in rainbow trout. *Development* **137**, 1227–1230 (2010). 20223765 10.1242/dev.044982

[28] Wong, T.-T., Saito, T., Crodian, J. & Collodi, P. Zebrafish germline chimeras produced by transplantation of ovarian germ cells into sterile host larvae. *Biol. Reprod.* **84**, 1190–1197 (2011). 21248287 10.1095/biolreprod.110.088427 PMC3099584

[29] Lee, S., Iwasaki, Y., Shikina, S. & Yoshizaki, G. Generation of functional eggs and sperm from cryopreserved whole testes. *Proc. Natl. Acad. Sci. U.S.A.* **110**, 1640–1645 (2013). 23319620 10.1073/pnas.1218468110 PMC3562789

[30] Lee, S., Katayama, N. & Yoshizaki, G. Generation of juvenile rainbow trout derived from cryopreserved whole ovaries by intraperitoneal transplantation of ovarian germ cells. *Biochem. Biophys. Res. Commun.* **478**, 1478–1483 (2016). 27581197 10.1016/j.bbrc.2016.08.156

[31] Nagoya, H., Okamoto, H., Nakayama, I., Araki, K. & Onozato, H. Production of Androgenetic Diploids in Amago Salmon *Oncorhynchus masou ishikawae*. *Fish. Sci.* **62**, 380–383 (1996).

[32] Komen, H. & Thorgaard, G. H. Androgenesis, gynogenesis and the production of clones in fishes: A review. *Aquaculture* **269**, 150–173 (2007).

[33] Babiak, I. et al. Androgenesis in rainbow trout using cryopreserved spermatozoa: the effect of processing and biological factors. *Theriogenology* **57**, 1229–1249 (2002). 12013444 10.1016/s0093-691x(02)00631-3

[34] Bercsényi, M., Magyary, I., Urbányi, B., Orbán, L. & Horváth, L. Hatching out goldfish from common carp eggs: interspecific androgenesis between two cyprinid species. *Genome* **41**, 573–579 (1998).

[35] Sato, M., Morita, T., Katayama, N. & Yoshizaki, G. Production of genetically diversified fish seeds using spermatogonial transplantation. *Aquaculture* **422–423**, 218–224 (2014).

[36] Octavera, A., Yamakawa, K. & Yoshizaki, G. The volume and shape of bitterling eggs are more strongly influenced by germ cell autonomy than by the surrounding somatic cells. *Fish Physiol. Biochem.* **49**, 967–981 (2023). 37667149 10.1007/s10695-023-01235-z

[37] Franěk, R., Kašpar, V., Shah, M. A., Gela, D. P. & M.,. Production of common carp donor-derived offspring from goldfish surrogate broodstock. *Aquaculture* **534**, 736252. 10.1016/j.aquaculture.2020.736252 (2021).

[38] Majhi, S. K. Generation of surrogate goldfish Carassius auratus progeny from common carp *Cyprinus carpio* parents. *3 Biotech*10.1007/s13205-022-03424-8 (2023). 36590242 10.1007/s13205-022-03424-8 PMC9794659

[39] Nayak, R., Franěk, R., Šindelka, R. & Pšenička, M. Enhancement of zebrafish sperm production via a large body-sized surrogate with germ cell transplantation. *Commun Biol* **6**, 412. 10.1038/s42003-023-04800-7 (2023). 37059808 10.1038/s42003-023-04800-7 PMC10104805

[40] Yoshikawa, H. et al. Induction of germ cell-deficiency in grass puffer by dead end 1 gene knockdown for use as a recipient in surrogate production of tiger puffer. *Aquaculture* **526**, 735385. 10.1016/j.aquaculture.2020.735385 (2020).

[41] Yoshizaki, G. et al. Gametes of semelparous salmon are repeatedly produced by surrogate rainbow trout. *Sci Adv*10.1126/sciadv.adm8713 (2024). 38787947 10.1126/sciadv.adm8713 PMC11122675

[42] Saitoh, K. et al. Natural habitats uncovered? - Genetic structure of known and newly found localities of the endangered bitterling *Pseudorhodeus tanago* (Cyprinidae). *Nat. Conserv.* **17**, 19–33 (2017).

[43] Kubota, H. et al. Genetic population structure and management units of the endangered Tokyo bitterling, *Tanakia tanago* (Cyprinidae). *Conserv. Genet.* **11**, 2343–2355 (2010).

[44] Octavera, A. & Yoshizaki, G. Production of donor-derived offspring by allogeneic transplantation of spermatogonia in Chinese rosy bitterling. *Biol. Reprod.* **100**, 1108–1117 (2019). 30544188 10.1093/biolre/ioy236

[45] Octavera, A. & Yoshizaki, G. Production of Chinese rosy bitterling offspring derived from frozen and vitrified whole testis by spermatogonial transplantation. *Fish Physiol. Biochem.* **46**, 1431–1442 (2020). 32356193 10.1007/s10695-020-00802-y

[46] Kise, K. et al. Flow-cytometric isolation and enrichment of teleost type A spermatogonia based on light-scattering properties. *Biol. Reprod.* **86**, 107 (2012). 22219211 10.1095/biolreprod.111.093161

[47] Takeuchi, Y., Higuchi, K., Yatabe, T., Miwa, M. & Yoshizaki, G. Development of spermatogonial cell transplantation in Nibe croaker, *Nibea mitsukurii* (Perciformes, Sciaenidae). *Biol. Reprod.* **81**, 1055–1063 (2009). 19605788 10.1095/biolreprod.109.077701

[48] Potthoff, T., Kelley, S., Moe, M. & Young, F. Description of porkfish larvae (*Anisotremus Virginicus*, Haemulidae) and their osteological development. *Bull. Mar. Sci.* **34**, 21–59 (1984).

