# Supplementary material for: Production of offspring via the transplantation of frozen germ cells from Tokyo bitterling, a fish on the brink of extinction
Source: Sci Rep. 2025 Nov 19;15:40759. doi: 10.1038/s41598-025-24449-y (PMC12630933; doi:10.1038/s41598-025-24449-y)
Supplement: Supplementary file 1 — Supplementary Information 1. [file 41598_2025_24449_MOESM1_ESM.pdf]

Fertilized date	Parent		No. of eggs	No. of haching larvae
	Male	Female		
2023.04.16	Rec. M#1	Rec. F#1	30	29
2023.04.21	Rec. M#1	Rec. F#1	16	13
2023.04.26	Rec. M#1	Rec. F#1	22	20
2023.06.03	Rec. M#1	Rec. F#2	18	16
2023.06.09	Rec. M#1	Rec. F#2	25	23
2023.06.10	Rec. M#1	Rec. F#2	33	29
2023.07.11	Rec. M#1	Rec. F#3	44	26
2023.07.31	Rec. M#1	Rec. F#4	35	25
2023.08.08	Rec. M#1	Rec. F#4	52	35
2023.08.16	Rec. M#1	Rec. F#4	47	42
2023.09.01	Rec. M#1	Rec. F#5	11	9
2023.09.10	Rec. M#1	Rec. F#5	36	30
2023.09.17	Rec. M#1	Rec. F#5	33	32
2023.06.16	Rec. M#2	Rec. F#1	39	35
2023.12.11	Rec. M#2	Rec. F#3	51	37
2023.09.29	Rec. M#2	Rec. F#5	43	43
2023.10.18	Rec. M#2	Rec. F#5	40	35
2023.07.12	Rec. M#3	Rec. F#1	51	34
2023.08.02	Rec. M#3	Rec. F#1	46	24
2023.08.07	Rec. M#3	Rec. F#1	48	37
2023.09.04	Rec. M#4	Rec. F#1	42	39
2023.12.11	Rec. M#4	Rec. F#2	36	23
2023.09.17	Rec. M#4	Rec. F#3	30	20
2023.10.28	Rec. M#4	Rec. F#5	33	30
2023.07.27	Rec. M#5	Rec. F#1	33	28
2023.11.03	Rec. M#5	Rec. F#3	35	29
2023.11.12	Rec. M#6	Rec. F#1	47	47
2023.11.10	Rec. M#6	Rec. F#3	40	39
2023.07.11	*Pt* M#1	*Pt F#1*	10	10
2023.07.20	*Pt* M#1	*Pt F#1*	7	7
2023.08.01	*Pt* M#1	*Pt F#1*	4	3
2023.07.10	*Pt M#2*	*Pt* F#2	9	9
2023.07.14	*Pt M#2*	*Pt* F#2	8	7
2023.07.24	*Pt M#2*	*Pt* F#2	9	9
2023.08.03	*Pt* M#3	*Pt* F#3	5	5
2023.08.09	*Pt* M#3	*Pt* F#3	6	6

2023.08.21	*Pt* M#3	*Pt* F#3	6	6
2023.10.29	*Tl* M#1	*Tl* F#1	43	40
2023.11.05	*Tl* M#1	*Tl* F#1	39	31
2023.11.12	*Tl* M#1	*Tl* F#1	43	37
2023.11.05	*Tl* M#2	*Tl* F#2	27	24
2023.11.12	*Tl* M#2	*Tl* F#2	29	26
2023.11.17	*Tl* M#2	*Tl* F#2	33	30
2023.11.16	*Tl* M#3	*Tl* F#3	35	30
2023.11.22	*Tl* M#3	*Tl* F#3	28	26
2023.11.29	*Tl* M#3	*Tl* F#3	34	31

Haching rate (%)
96.7
81.3
90.9
88.9
92.0
87.9
59.1
71.4
67.3
89.4
81.8
83.3
97.0
89.7
72.5
100.0
87.5
66.7
52.2
77.1
92.9
63.9
66.7
90.9
84.8
82.9
100.0
97.5
100.0
100.0
75.0
100.0
87.5
100.0
100.0
100.0

100.0

93.0
79.5
86.0
88.9
89.7
90.9
85.7
92.9
91.2
