# Supplementary material for: Production of offspring via the transplantation of frozen germ cells from Tokyo bitterling, a fish on the brink of extinction
Source: Sci Rep. 2025 Nov 19;15:40759. doi: 10.1038/s41598-025-24449-y (PMC12630933; doi:10.1038/s41598-025-24449-y)
Supplement: Supplementary file 2 — Supplementary Information 2. [file 41598_2025_24449_MOESM2_ESM.pdf]

Fig. 1E

cell_membrane_permeating_cryoprotectants

	DMSO	PG	Gly	EG	MeOH
no.1	49.45	36.36	37.11	17.95	29.00
no.2	43.90	33.33	32.26	50.00	30.56
no.3	50.00	36.84	28.57	31.43	8.00
no.4	50.00	32.14	36.00	32.56	22.58
no.5	57.14	29.41	26.32	19.23	21.21

Fig. 1F

DMSO_concentration

	1.0 M	1.3 M	1.6 M
no.1	46.97	52.38	43.66
no.2	50.82	55.32	40.00
no.3	47.27	52.24	39.39
no.4	45.83	55.56	45.71
no.5	47.37	51.85	38.46

Fig. 1G

cell_membrane_impermeating_cryoprotectants

	0.1 M	0.2 M	0.05 M	0.1 M	0.05 M	0.1 M
no.1	40.91	47.62	47.92	48.28	43.28	39.73
no.2	37.93	45.07	40.26	45.19	34.35	48.86
no.3	34.09	45.63	43.30	45.95	39.13	37.31
no.4	43.59	52.17	53.85	53.33	44.90	52.78
no.5	46.15	45.10	41.38	50.00	45.61	51.35

Fig. 1H

equilibration_time

	**15 min**	**30 min**	**45 min**
no.1	48.98	40.00	43.33
no.2	49.15	34.04	32.94
no.3	53.70	41.07	35.42
no.4	50.00	45.61	41.18
no.5	57.14	42.11	42.86

Fig. 4D

Length, width, and volume of unfertilized eggs produced by Tokyo bitterling, recipient and oily bitterling.

Recipient female① (mm)

	①			②			③		
	egg width	egg length	egg volume	egg width	egg length	egg volume	egg width	egg length	egg volume
1	1327.87	2130.43	1966871863.85	1415.34	2181.54	2288146135.66	1461.68	2219.09	2482433043.19
2	1342.35	2093.44	1975102322.10	1490.39	2199.31	2557902985.32	1418.83	2100.17	2213680118.78
3	1400.29	2288.08	2349127633.83	1492.16	2172.94	2533259094.93	1424.85	2167.94	2304547779.41
4	1391.12	2240.04	2269776060.01	1433.93	2158.55	2323905332.74	1368.73	2005.21	1966942541.39
5	1360.64	2134.20	2068808638.11	1444.06	2165.45	2364368182.42	1408.78	2113.17	2195919563.49
6	1404.32	2165.90	2236502686.54	1435.16	2224.81	2399359001.11	1453.74	2152.37	2381719742.30
7	1461.68	2195.09	2455584497.47	1413.84	2200.40	2303043477.31	1437.62	2115.13	2288888914.06
8	1408.78	2099.50	2181720438.49	1461.68	2227.04	2491318260.91	1451.74	2224.81	2455100646.14
9	1383.80	2228.90	2234788541.72	1520.18	2106.43	2548804888.73	1438.36	2182.79	2364532545.17
10	1387.31	2097.02	2113223701.17	1437.62	2103.07	2275843092.25	1462.22	2101.01	2352090270.76
11	1409.53	2184.57	2272544418.55	1475.92	2167.41	2472104916.47	1448.39	2232.42	2452142322.97
12	1418.89	2213.52	2333352792.24	1467.83	2167.73	2445421012.50	1497.72	2109.61	2477761713.56
13	1344.45	1967.74	1862335058.84	1396.69	2181.90	2228616900.94	1441.24	2161.86	2351253660.98
14	1411.59	2147.64	2240683303.08	1430.73	2095.71	2246178949.34	1457.14	2180.61	2424258704.82

15	1433.38	2045.70	2200709659.45	1452.23	2145.30	2368941637.30	1468.97	2144.48	2422950975.31
16	1384.63	2161.86	2170172751.69	1172.06	1855.23	1334426446.03	1435.10	2188.33	2359804702.80
17	1342.09	2048.46	1931912083.37				1455.93	2166.31	2404357346.24
18	1403.25	2102.40	2167628799.02				1443.93	2187.52	2388057087.34
19	1289.21	1930.05	1679631891.54				1470.71	2044.15	2315074892.97
20	1441.30	2133.04	2320106866.39				1356.48	2158.10	2079207329.90
21	1364.01	2133.08	2077980485.62				1420.32	2288.08	2416819740.34
22	1422.56	2166.31	2295405209.93				1476.64	2166.59	2473579226.11
23	1438.17	2122.92	2299086385.19						
24	1432.95	2207.29	2373108161.58						
25	1375.03	2147.93	2126402425.18						
26	1421.07	2177.32	2302246813.36						
27	1444.06	2195.41	2397082931.16						
28	1409.90	2195.25	2284875726.22						
29	1455.75	2169.24	2407010202.32						
30	1400.25	2184.56	2242717088.79						

	④			⑤			⑥		
	egg width	egg length	egg volume	egg width	egg length	egg volume	egg width	egg length	egg volume
1	1475.21	2147.64	2447179593.31	1447.11	2127.82	2333116037.24	1441.24	2242.48	2438937719.10
2	1510.86	2187.96	2615097125.35	1486.00	2259.46	2612406191.66	1441.30	2175.42	2366198827.93
3	1445.89	2004.68	2194388573.71	1396.82	2246.42	2294928145.19	1514.83	2152.65	2586428154.77
4	1486.00	2123.96	2455732622.10	1582.33	2245.83	2944211786.75	1340.05	2203.76	2072060727.79
5	1455.87	2176.35	2415302628.73	1505.83	2266.95	2691486274.02	1449.85	2324.03	2557924616.61
6	1483.80	2179.43	2512415934.33	1503.66	2270.07	2687417454.83	1462.64	2114.84	2368931820.01
7	1492.64	2082.53	2429394749.54	1605.37	2337.67	3154516963.31	1505.24	2283.72	2709275966.10
8	1501.60	2273.22	2683788473.68	1613.93	2319.66	3163671172.93	1551.06	2040.69	2570574252.34
9	1491.87	2147.27	2502343561.76	1457.14	2164.27	2406096500.43	1458.84	2266.02	2525082236.51
10	1490.21	2080.28	2418886879.26	1244.25	1944.41	1576176544.83	1432.27	2251.44	2418290906.97
11	1487.90	2143.03	2484128992.58	1412.53	2170.99	2268051118.46	1448.45	2033.76	2234112558.17
12	1520.18	2159.86	2613457216.72	1485.94	2167.41	2505768986.02	1354.92	2240.04	2153180811.67
13	1297.13	1841.72	1622507493.83	1307.63	1795.11	1607170370.08	1456.41	2283.25	2535841658.95
14	1491.16	2145.30	2497662595.32	1517.97	2368.77	2857916224.72	1438.30	2267.89	2456506132.94
15	1461.68	2086.38	2333973711.67	1475.03	2259.00	2573435818.48	1507.59	2060.71	2452332046.36
16	1511.45	2182.18	2610208925.61	1545.07	2170.87	2713480347.07	1512.79	2273.91	2724767256.08

17	1515.06	2059.68	2475482101.74	1485.64	2152.57	2487620806.18	1525.05	2160.72	2631275742.86
18	1475.21	2150.52	2450456294.24	1573.71	2275.35	2950522463.85	1450.58	2222.31	2448436665.66
19	1492.64	2208.92	2576843846.56	1399.79	2140.03	2195539015.19	1466.56	2120.96	2388541485.20
20	1451.07	2144.68	2364492569.08	1415.34	2201.96	2309566484.61	1520.65	2117.84	2564178912.09
21	1506.94	2213.84	2632307995.34	1507.35	2213.04	2632791683.60	1412.53	2155.15	2251506984.32
22	1485.94	2137.42	2471104917.41	1549.63	2162.68	2719239072.74	1415.28	2178.99	2285268960.57
23	1524.01	2155.15	2620913528.48	1548.04	2220.33	2785975288.38	1536.13	2071.60	2559528570.19
24	1466.26	2116.63	2382682917.16	1289.00	1886.89	1641548315.42	1535.04	2072.12	2556520487.75
25	1523.26	2201.84	2675042332.51	1480.64	2159.08	2478378280.37	1375.03	2280.12	2257264591.07
26	1486.65	2192.32	2536997356.10	1537.85	2176.15	2694730781.80	1429.31	2229.25	2384567867.24
27	1507.41	2218.73	2639774854.09	1463.19	2218.62	2487026808.92	1538.02	2096.13	2596231145.93
28	1260.33	1757.68	1461863910.39	1477.12	2372.57	2710490596.98	1382.21	2101.56	2102257227.69
29	1505.83	2161.17	2565887579.86	1544.21	2260.75	2822698999.85	1391.18	2115.13	2143401229.14
30	1468.97	2182.35	2465738817.75	1596.00	2259.19	3013114455.40	1445.16	2205.97	2412273936.07
31	1510.16	2015.09	2406242154.87	1462.22	2233.37	2500262000.69	1545.07	2170.87	2713480347.07
32	1472.15	2186.06	2480651962.20	1550.37	2257.00	2840550111.16	1461.74	2219.13	2482682711.82
33	1510.86	2213.84	2646021130.84	1488.85	2155.15	2501364485.06	1444.24	2128.11	2324187305.10
34	1485.64	2020.17	2334607490.55	1533.88	2243.11	2763343481.33	1528.18	2152.82	2632402708.75

35	1461.98	2204.40	2467019605.75	1582.61	2132.21	2796255120.09	1445.89	2177.69	2383769392.18
36	1496.30	2062.85	2418262697.53	1603.89	2275.31	3064696188.70	1486.47	2100.05	2429640339.11
37	1448.45	2086.59	2292156013.33	1527.42	2188.93	2673930113.46	1435.10	2152.69	2321381406.09
38	1407.84	2201.88	2285054180.37	1563.81	2146.33	2748289729.68	1480.94	2200.40	2526817686.75
39	1489.02	2196.54	2550008813.81	1533.88	2160.43	2661487081.40	1500.19	2183.28	2572760301.62
40				1328.86	2152.82	1990523173.31	1501.78	2298.86	2714700810.26
41				1334.76	1738.90	1622116934.63	1275.92	1847.46	1574789643.54
42				1258.44	1742.09	1444549400.85	1592.62	2196.18	2916687391.99
43				1443.93	2050.36	2238322312.48	1440.08	2180.61	2367810269.28
44				1485.23	2215.31	2558691748.18	1529.91	1962.71	2405391174.50
45				1470.11	2135.44	2416493844.01	1503.66	2061.74	2440784366.07
46				1465.90	2222.91	2501087788.63	1392.64	2323.12	2359111275.01
47				1496.42	2090.65	2451241745.01			
48				1486.00	2207.29	2552075902.37			
49				1614.37	2192.32	2991617877.76			
50				1387.88	2089.93	2107823092.90			
51				1477.60	2283.06	2609924830.54			

	⑦			⑧			⑨		
	egg width	egg length	egg volume	egg width	egg length	egg volume	egg width	egg length	egg volume
1	1357.78	2174.12	2098659508.44	1441.30	2312.45	2515251533.62	1494.23	2546.48	2976976715.90
2	1457.14	2238.27	2488361171.86	1477.90	2276.13	2603052080.20	1522.16	2408.98	2922463937.91
3	1443.08	2233.01	2434832957.13	1503.66	2248.18	2661509572.05	1513.31	2510.16	3009944782.96
4	1400.04	2123.50	2179371392.97	1511.10	2329.49	2785119768.52	1424.48	2558.10	2717877548.14
5	1420.88	2145.34	2267833076.92	1368.66	2033.06	1994075429.18	1527.83	2511.25	3069294521.92
6	1415.34	2121.59	2225267151.45	1458.65	2254.50	2511616973.75	1483.02	2501.00	2880111033.90
7	1379.46	2043.80	2036354664.55	1504.42	2223.66	2635154018.06	1485.64	2472.14	2856926467.35
8	1353.16	2104.79	2017923922.87	1533.88	2209.68	2722162497.18	1491.63	2501.85	2914625147.89
9	1450.03	1890.49	2081279997.45	1468.25	2288.66	2583318606.74	1481.65	2419.00	2780531936.58
10	1364.59	2104.08	2051476844.24	1519.31	2220.52	2683783966.74	1516.98	2529.75	3048162796.27
11	1435.16	2073.61	2236288150.73	1436.09	2328.55	2514457565.09	1452.29	2439.82	2694390610.19
12	1436.15	2032.67	2195147233.13	1402.56	2124.25	2187994953.18	1444.48	2516.49	2749276089.84
13	1473.05	2230.20	2533832843.04	1473.05	2288.20	2599719938.35	1491.63	2342.65	2729155021.67
14	1385.84	2244.73	2257298474.68	1479.21	2181.05	2498756648.46	1488.31	2482.40	2879115456.88
15	1445.52	2025.53	2216092841.17	1447.48	2392.91	2625105877.34	1504.30	2472.60	2929699599.78
16	1389.66	2129.44	2153177730.87	1436.09	2253.95	2433904706.77	1503.66	2550.63	3019566268.23

17	1419.08	2189.29	2308425773.62	1500.54	2261.30	2665957887.05	1521.40	2573.69	3119190880.62
18	1434.24	2133.54	2297963275.64	1484.69	2161.82	2495111885.30	1466.80	2517.92	2836512806.93
19	1396.25	2025.80	2067858704.04	1449.91	2222.31	2446176633.49	1545.35	2523.35	3155236074.56
20	1462.04	1854.85	2075996306.97	1468.79	2288.81	2585397217.36	1437.62	2574.58	2786084307.54
21	1401.55	2175.50	2237564309.94	1522.04	2276.01	2760735431.90	1489.02	2497.93	2899897044.96
22	1482.19	2135.40	2456326332.79	1552.19	2277.52	2873114696.21	1536.41	2550.53	3152435607.79
23	1415.34	2236.96	2346281547.81	1464.45	2283.14	2563786561.00	1440.08	2446.18	2656183655.46
24	1357.72	2166.59	2091195005.31	1466.80	2236.49	2519470005.19	1460.29	2560.86	2859312116.42
25	1430.97	2290.66	2455974432.01	1522.97	2244.88	2726298687.68	1475.21	2471.89	2816644631.68
26	1455.75	2205.97	2447760549.60	1449.85	2227.47	2451648824.10	1515.06	2365.41	2842931980.54
27	1443.08	2297.28	2504912257.47	1474.73	2313.86	2634872259.13	1438.54	2555.99	2769517895.18
28	1334.76	2104.79	1963438031.03	1467.71	2107.73	2377337555.11	1481.71	2632.26	3025914121.18
29	1387.12	2247.83	2264577126.74	1500.13	2269.48	2674137635.95	1493.05	2528.53	2951313566.54
30	1361.48	2126.49	2063892437.11	1555.83	2272.91	2880738230.26	1461.92	2478.63	2773682820.04
31	1448.63	2291.24	2517601276.41	1541.46	2285.69	2843686782.51	1492.16	2447.66	2853531810.71
32	1445.46	2167.41	2371112707.84	1522.27	2274.54	2759788075.75	1424.48	2557.17	2716887341.93
33	1409.40	2198.67	2286804741.34	1506.30	2272.91	2700232420.95	1568.88	2420.20	3119111142.08
34	1422.81	2292.36	2429816746.65				1557.87	2462.55	3129290515.83

35	1356.42	2158.10	2079007808.99				1532.96	2377.36	2925210881.29
36	1413.84	2080.96	2178035732.19				1452.95	2552.57	2821504037.54
37	1451.07	1972.00	2174113380.35				1477.12	2462.62	2813369866.07
38	1405.89	2116.25	2190129912.64				1507.41	2546.89	3030207347.88
39	1385.65	2092.80	2103946277.67				1461.98	2437.18	2727523425.88
40	1440.08	2292.05	2488823530.85				1453.93	2562.14	2835864144.91
41	1433.75	2171.84	2337613774.66				1463.61	2532.54	2840566344.19
42	1358.04	2219.69	2143469844.03				1497.95	2466.88	2898291409.13
43	1460.29	2047.94	2286620025.75				1479.75	2543.67	2916311072.06
44	1386.03	2270.22	2283569351.61				1429.00	2534.91	2710343285.10
45	1445.89	2198.15	2406163478.15				1545.07	2545.82	3182152023.48
46	1449.85	2269.10	2497461842.21				1420.39	2589.55	2735495261.17
47	1442.04	2206.17	2402090554.09				1509.46	2466.88	2942992787.48
48	1374.84	2181.82	2159346402.75						

Recipient female② (mm)

	①		
	egg width	egg length	egg volume
1	1479.33	2303.46	2639425644.40
2	1459.50	2288.08	2551992415.53
3	1416.59	2282.17	2397916555.49
4	1493.29	2442.97	2852351316.39
5	1522.62	2300.20	2792206727.03
6	1495.41	2653.94	3107512714.46
7	1541.46	2378.70	2959402881.49
8	1311.28	2319.66	2088383262.06
9	1413.84	2238.27	2342680817.18
10	1479.81	2376.02	2724326298.85
11	1310.20	2123.50	1908643246.78
12	1494.17	2448.78	2862532485.84
13	1386.03	2381.81	2395814569.32
14	1486.83	2253.56	2608492376.53
15	1550.60	2348.97	2957162576.27
16	1396.50	2294.21	2342691628.86
17	1156.05	1954.46	1367674091.28
18	1424.79	2182.02	2319318785.65
19	1533.88	2337.67	2879825879.43
20	1543.47	2223.90	2774013618.89
21	1785.64	2273.91	3796300216.83
22	1493.29	2379.59	2778352682.73
23	1517.97	2439.35	2943073878.22
24	1645.35	2497.65	3540351081.26
25	1424.54	2428.90	2580835625.52
26	1488.37	2290.97	2657308133.43
27	1503.48	2320.84	2746875902.76
28	1503.66	2355.72	2788820526.12
29	1488.31	2365.30	2743299493.72
30	1517.51	2381.22	2871179453.56
31	1473.77	2474.17	2813758227.75

32	1362.39	2367.09	2300470243.42
33	1448.94	2339.63	2571848654.76
34	1473.11	2460.32	2795507489.59
35	1529.56	2472.85	3029216868.95
36	1423.37	2355.01	2498184536.01

Recipient female③ (mm)

	①			②			③		
	egg width	egg length	egg volume	egg width	egg length	egg volume	egg width	egg length	egg volume
1	1448.63	2339.63	2570767137.24	1453.93	2286.15	2530394679.00	1488.37	2108.48	2445634767.56
2	1412.78	2433.15	2542834828.51	1441.12	2322.66	2525711880.32	1317.39	2274.15	2066544315.65
3	1422.56	2415.23	2559166347.08	1256.12	2144.31	1771526653.29	1468.25	2127.74	2401678145.34
4	1394.67	2352.24	2395639186.88	1448.45	2272.24	2496096437.22	1447.48	2199.43	2412856816.56
5	1505.83	2330.90	2767401141.02	1424.05	2328.47	2472397662.29	1470.23	2217.82	2510124422.41
6	1444.18	2499.31	2729352575.22	1410.84	2321.86	2419874459.42	1457.14	2267.73	2521121372.05
7	1410.84	2430.97	2533591405.99	1474.73	2255.55	2568472279.99	1441.30	2016.97	2193859802.97
8	1406.08	2375.02	2458587242.93	1441.24	2423.55	2635873001.03	1395.24	2205.85	2248382872.01
9	1424.05	2363.69	2509797397.29	1488.49	2325.74	2698062874.07	1382.01	2414.80	2414926653.38
10	1475.50	2421.84	2760740071.23	1462.64	2378.88	2664704059.53	1313.97	2509.14	2268255880.29
11	1400.04	2422.13	2485860966.30	1456.66	2346.71	2607188839.08	1431.16	2272.59	2437230623.80
12	1447.48	2480.62	2721331427.53	1479.81	2252.73	2582964077.73	1507.41	2111.95	2512729695.35
13	1472.15	2480.62	2814901562.58	1446.38	2362.50	2587813626.62	1418.33	2301.74	2424435390.10
14	1434.67	2430.10	2618955375.81	1489.20	2266.95	2632383398.36	1436.64	2274.73	2458237975.33
15	1455.75	2461.00	2730754859.32	1513.02	2327.33	2789636312.69	1508.58	2197.99	2619153240.38

16	1432.27	2284.80	2454121248.85	1441.24	2243.78	2440350318.18	1520.65	2217.34	2684651885.87
17	1425.53	2504.95	2665346481.86	1429.31	2287.58	2446956117.44	1426.53	2371.11	2526445798.09
18	1442.53	2472.60	2694016056.76	1470.11	2313.98	2618529823.01	1477.60	2335.06	2669368114.25
19	1415.34	2462.26	2582587545.56	1444.06	2457.85	2683620978.92	1428.20	2209.04	2359273727.29
20	1455.93	2413.92	2679177062.70	1503.89	2329.61	2758764325.66	1464.63	2277.83	2558462651.71
21	1426.53	2456.88	2617826020.52	1569.16	2245.83	2895418432.03	1437.62	2264.46	2450489824.10
22	1441.30	2410.37	2621754256.24	1492.05	2346.07	2734665652.34	1427.76	2112.96	2255283373.52
23	1466.56	2468.63	2780073592.99	1466.56	2467.88	2779227698.16	1403.88	2433.59	2511341210.98
24	1424.05	2410.08	2559046915.60	1486.83	2266.64	2623639361.63	1459.50	2322.05	2589883418.03
25	1483.80	2384.18	2748451335.55	1524.65	2330.52	2836545347.50	1489.20	2329.19	2704651337.12
26	1419.08	2443.04	2575978241.09	1355.63	2247.08	2162236644.12	1479.81	2208.56	2532319925.97
27	1463.91	2427.19	2723532853.73	1528.18	2378.70	2908602401.02	1474.73	2071.22	2358565995.55
28	1469.69	2426.18	2743923095.97	1428.32	2366.60	2527985941.78	1485.94	2065.16	2387557991.66
29	1373.88	2510.87	2481520834.79	1477.06	2349.01	2683357966.19	1406.08	2242.48	2321386062.63
30	1435.16	2470.03	2663810492.12	1461.98	2285.57	2557857126.44	1363.23	2292.63	2230861635.80
31	1448.39	2471.56	2714823763.76	1396.82	2413.04	2465151144.57			
32	1470.17	2473.14	2798862359.56	1434.24	2405.46	2590835552.66			
33	1463.19	2424.43	2717739483.10	1465.12	2417.76	2717411906.87			

34	1463.49	2326.91	2609504122.79	1507.41	2359.02	2806680250.53			
35	1475.92	2434.64	2776904455.57	1422.87	2306.95	2445493246.97			
36	1463.19	2555.89	2865106065.17	1451.74	2313.86	2553368132.25			
37	1356.42	2601.80	2506444817.08	1489.20	2233.80	2593889195.23			
38	1489.02	2443.91	2837180187.78	1473.11	2270.65	2579995868.91			
39	1431.16	2406.56	2580896644.87	1476.46	2303.46	2629203566.71			
40	1447.17	2436.45	2671750272.88	1474.73	2221.96	2530212477.80			
41	1475.92	2445.31	2789078941.80	1497.72	2264.50	2659680367.77			
42	1440.51	2488.26	2703487751.95	1494.00	2320.84	2712330751.98			
43	1501.60	2298.86	2714063207.31	1411.59	2449.75	2555877708.16			
44	1444.06	2577.90	2814706390.23	1461.74	2378.55	2661031367.23			
45				1467.71	2343.85	2643665339.45			
46				1438.11	2338.23	2532050567.07			
47				1458.65	2357.22	2626057433.13			
48				1452.95	2345.02	2592082402.56			
49				1501.78	2292.71	2707434953.64			
50				1472.57	2363.95	2684042754.57			
51				1527.66	2362.50	2886827540.76			

	④			⑤		
	egg width	egg length	egg volume	egg width	egg length	egg volume
1	1433.19	2214.79	2381997861.73	1440.08	2418.89	2626547849.09
2	1457.14	2175.50	2418580748.50	1414.78	2389.03	2503791082.11
3	1441.30	2105.42	2290064667.94	1371.82	2451.08	2415185456.32
4	1420.51	2189.29	2313081082.06	1400.04	2358.57	2420625179.81
5	1468.01	2213.04	2497141941.12	1405.33	2361.67	2442152360.14
6	1465.78	2377.21	2674261171.89	1426.53	2294.94	2445280605.08
7	1448.45	2152.69	2364767992.73	1395.24	2285.57	2329646825.19
8	1419.08	2223.39	2344373157.77	1443.51	2261.30	2467139980.01
9	1425.78	2283.45	2430501705.21	1441.12	2351.48	2557054704.46
10	1457.14	2381.66	2647782790.94	1418.89	2245.20	2366748927.18
11	1390.23	2251.01	2277977313.65	1410.09	2288.66	2382730021.33
12	1658.66	2375.02	3421206379.57	1426.28	2317.22	2468164427.04
13	1411.59	2189.25	2284095134.55	1436.15	2263.06	2443949123.40
14	1412.53	2277.64	2379468945.43	1426.53	2313.52	2465080440.70
15	1402.50	2115.63	2178920116.76	1448.63	2338.84	2569896274.12
16	1435.66	2112.96	2280287814.56	1411.91	2332.22	2434334479.77
17	1432.27	2253.20	2420185509.15	1333.37	2246.97	2091700495.27
18	1409.09	2225.77	2313959629.76	1391.12	2236.57	2266259008.08
19	1396.31	2241.42	2288165729.16	1435.16	2046.39	2206938961.87
20	1384.63	2229.73	2238302241.89	1378.37	2377.66	2365256754.03
21	1438.11	2192.88	2374649747.70	1434.24	2384.22	2567964981.96
22	1422.81	2261.46	2397060693.15	1424.54	2298.97	2442779302.00
23	1392.70	2249.75	2284818656.32	1454.29	2391.54	2648371420.37
24	1422.06	2261.30	2394386424.12	1408.78	2325.74	2416817556.95
25	1424.30	2187.72	2323756664.84	1422.75	2293.09	2430380242.91
26	1466.56	2253.36	2537642468.90	1376.38	2391.58	2372250423.07
27	1413.41	2381.22	2490762485.63	1409.90	2264.93	2357395435.72
28	1370.79	2265.55	2229020191.31	1382.01	2377.36	2377489375.83
29	1450.03	2256.02	2483700277.98	1357.00	2528.94	2438364279.44
30	1419.51	2222.59	2344974024.57	1317.59	2248.03	2043430981.59
31	1412.91	2286.03	2389508222.66	1420.76	2298.36	2429164571.49
32	1374.58	2315.35	2290646654.53	1474.19	2241.89	2551048900.64

33	1429.99	2359.32	2526092859.29	1374.07	2366.04	2339048072.25
34	1448.45	2219.13	2437751919.83	1383.80	2340.27	2346454591.24
35	1352.57	2303.46	2206478062.10	1400.29	2377.21	2440636942.30
36				1411.91	2290.20	2390476189.55
37				1380.22	2443.62	2437422719.70
38				1409.09	2485.42	2583902373.44
39				1445.16	2322.66	2539884383.65
40				1506.30	2351.71	2793853131.80

Recipient female④

	①			②			③		
	egg width	egg length	egg volume	egg width	egg length	egg volume	egg width	egg length	egg volume
1	1655.94	2336.87	3355236024.13	1507.41	2309.89	2748231804.49	1427.33	2363.39	2521060638.14
2	1622.49	2499.87	3445743176.08	1514.83	2551.43	3065562635.47	1514.19	2230.20	2677339040.36
3	1533.60	2437.94	3002226359.36	1449.85	2496.51	2747768566.74	1482.55	2424.10	2789760625.76
4	1537.97	2418.63	2995446441.14	1542.44	2214.15	2758170339.44	1513.02	2170.99	2602237411.57
5	1419.51	2187.52	2307969806.97	1494.17	2497.65	2919658170.59	1483.80	2345.02	2703301729.47
6	1554.92	2445.13	3095407189.78	1536.93	2356.51	2914588308.61	1475.21	2496.23	2844385519.95
7	1411.59	2201.56	2296933594.83	1493.05	2362.50	2757524226.54	1466.80	2336.57	2632212687.34
8	1556.11	2355.01	2985890075.54	1463.61	2359.80	2646824695.24	1531.81	2273.84	2793626691.84
9	1506.94	2333.39	2774466329.76	1493.58	2544.78	2972402080.06	1414.90	2425.88	2542864953.55
10	1551.11	2264.19	2852315242.82	1459.80	2535.64	2829275376.12	1465.78	2435.80	2740169744.37
11	1492.16	2360.67	2752112393.98	1533.88	2446.50	3013907764.47	1515.65	2194.17	2639147806.36
12	1533.88	2476.31	3050626642.18	1552.42	2462.40	3107253466.10	1473.47	2286.92	2599752973.12
13	1568.04	2429.05	3127145848.11	1465.12	2477.84	2784947125.39	1485.64	2489.96	2877528827.95
14	1475.92	2564.61	2925151777.33	1485.64	2441.85	2821920763.13	1479.21	2336.34	2676669239.51
15	1488.49	2432.03	2821367446.79	1475.92	2556.82	2916264662.25	1582.16	2207.25	2893021305.52
16	1487.54	2306.34	2672142509.15	1555.32	2266.02	2870125830.93	1426.53	2414.80	2572989708.81

17	1541.92	2392.17	2977932675.70	1460.77	2452.81	2740485719.49	1488.49	2379.88	2760877160.62
18	1470.89	2463.69	2790909833.09	1541.41	2391.87	2975574905.91	1475.44	2351.71	2680577063.19
19	1420.39	2492.09	2632537871.96	1485.94	2413.04	2789747969.49	1461.68	2445.02	2735176201.43
20	1536.93	2380.89	2944736686.35	1596.61	2408.39	3214551185.00	1489.02	2436.78	2828906365.00
21	1467.46	2513.68	2834285718.37	1515.06	2284.99	2746279382.83	1515.35	2380.92	2862677309.21
22	1488.85	2405.71	2792173666.32	1489.20	2486.13	2886890971.89	1449.36	2341.10	2574980946.50
23	1598.98	2366.38	3167887678.68	1442.53	2479.41	2701436264.87	1497.95	2038.31	2394770345.92
24	1441.24	2488.08	2706055338.84	1471.67	2470.42	2801500217.65	1517.68	2166.59	2612992226.46
25	1626.14	2290.66	3171567913.57	1492.16	2461.43	2869591872.78	1444.24	2406.74	2628487566.19
26	1396.82	2301.93	2351640752.79	1531.81	2392.57	2939507067.05	1433.93	2294.52	2470285604.19
27	1628.96	2388.03	3317859415.31	1455.75	2513.68	2789201147.25	1448.45	2329.49	2558984928.05
28	1385.08	2274.15	2284363006.36	1445.52	2464.02	2695825526.22	1459.14	2309.89	2575039171.54
29	1466.80	2439.82	2748526545.43	1493.29	2352.01	2746153838.86	1498.95	2430.07	2858858104.95
30	1559.17	2274.54	2895211995.34	1512.85	2385.37	2858539396.74	1480.28	2359.47	2707089296.52
31	1446.44	2393.90	2622436003.96	1559.85	2358.42	3004602953.94	1461.68	2484.89	2779770158.99
32	1504.42	2330.40	2761645666.21	1483.14	2383.70	2745472186.57	1485.64	2288.08	2644222910.66
33	1526.90	2466.34	3010757873.37	1494.71	2425.34	2837151980.54	1531.41	2358.42	2896018609.16
34	1531.52	2455.15	3015251942.50	1520.65	2415.71	2924826441.66	1526.04	2219.69	2706579585.73

35	1514.19	2345.39	2815623386.01	1557.02	2304.92	2925788303.62	1435.72	2437.18	2630409621.96
36	1477.60	2371.45	2710968934.79	1506.30	2443.44	2902823844.87	1462.46	2418.74	2708680613.49
37	1485.64	2365.41	2733588442.83	1457.14	2459.39	2734192653.00	1471.31	2424.57	2748163945.10
38	1517.92	2384.18	2876296533.61	1504.54	2416.26	2863834988.17	1475.44	2509.18	2860065340.55
39	1520.18	2237.75	2707709704.86	1517.97	2442.46	2946826742.96	1570.51	2040.00	2634581255.07
40	1491.16	2380.92	2771987339.86	1532.79	2395.52	2946896626.22	1477.06	2454.29	2803625001.33
41	1514.19	2405.57	2887857470.43	1527.42	2440.98	2981822951.12	1463.91	2366.04	2654916903.85
42	1643.04	2273.95	3214233304.87	1494.17	2416.00	2824219691.71	1435.66	2392.76	2582249368.35
43	1594.56	2519.68	3354469750.72	1510.86	2380.37	2845063106.45	1451.37	2447.59	2699574240.08
44	1648.19	2328.89	3312551213.68	1538.77	2363.69	2930464353.30	1448.63	2564.61	2817980779.25
45	1586.84	2361.56	3113623686.62	1495.00	2376.92	2781604779.81	1496.77	2447.66	2871182522.94
46	1686.32	2306.34	3433997375.51	1553.39	2382.44	3010097676.66	1513.02	2444.88	2930534264.89
47				1498.95	2541.38	2989809178.15	1479.03	2462.04	2819998365.64
48				1529.68	2281.36	2795061724.91			
49				1450.77	2576.16	2838997512.99			
50				1646.91	2352.69	3341181823.87			
51				1475.03	2507.88	2856958053.25			
52				1552.19	2516.87	3175054977.08			

Recipient female⑤ (mm)

	①			②			③		
	egg width	egg length	egg volume	egg width	egg length	egg volume	egg width	egg length	egg volume
1	1445.46	2600.92	2845364956.86	1470.17	2630.48	2976933611.62	1530.37	2714.30	3328498670.69
2	1437.31	2690.38	2910156970.66	1478.08	2588.43	2960928999.21	1508.64	2669.16	3180863313.25
3	1471.31	2681.54	3039424686.51	1473.11	2471.49	2808199614.47	1512.79	2753.18	3299055324.14
4	1472.15	2652.61	3010065761.23	1489.20	2779.22	3227227278.42	1582.33	2612.70	3425175726.88
5	1468.97	2600.98	2938740197.60	1450.28	2617.43	2882546019.64	1607.90	2498.18	3381739089.04
6	1480.28	2621.10	3007270665.93	1490.21	2488.30	2893316056.15	1558.32	2674.98	3401219471.70
7	1462.40	2602.04	2913707388.05	1488.49	2604.75	3021740393.28	1555.60	2531.91	3208070793.98
8	1456.84	2538.32	2820766266.79	1488.49	2578.42	2991191630.67	1528.12	2612.47	3194206477.42
9	1457.14	2707.95	3010521634.77	1501.13	2611.28	3080983208.17	1552.65	2653.61	3349510680.92
10	1483.02	2742.25	3157933809.66	1483.32	2614.90	3012480963.52	1533.88	2643.11	3256105776.01
11	1515.65	2614.90	3145203220.88	1489.02	2526.81	2933430526.47	1541.06	2597.52	3229965405.39
12				1496.06	2678.28	3138734777.68	1522.04	2582.83	3132898042.70
13				1467.71	2669.56	3011039769.11	1485.46	2615.94	3022393074.24
14				1523.26	2644.44	3212766229.04	1331.78	2640.43	2452119969.95
15				1475.92	2612.70	2979999502.29	1517.68	2611.08	3149059990.54
16				1453.93	2605.26	2883593909.88	1535.73	2630.25	3248047606.04

17				1483.56	2600.92	2997335771.76	1527.25	2579.51	3150337023.33
18				1400.48	2727.28	2800796338.36	1541.63	2617.73	3257519228.97
19				1477.90	2605.97	2980270524.28	1569.67	2681.70	3459603232.32
20				1520.18	2511.74	3039243498.88	1558.38	2590.37	3293881331.25
21				1495.41	2590.20	3032882257.85	1507.41	2568.74	3056201973.06
22				1493.05	2452.27	2862309943.76	1590.68	2668.54	3535379207.26
23				1438.30	2608.85	2825821489.66	1537.33	2552.54	3158691855.20
24				1480.28	2590.10	2971700194.24	1533.60	2585.70	3184189510.74
25				1498.95	2555.79	3006762596.55	1514.89	2604.48	3129540272.91
26				1449.36	2751.13	3025969718.11	1500.60	2588.67	3052146639.40
27				1476.46	2726.66	3112246359.87	1505.30	2679.33	3178861167.52
28				1490.92	2425.41	2822886145.26	1528.18	2697.75	3298736894.13
29				1483.02	2596.74	2990359792.39	1516.52	2585.70	3113668797.10
30				1501.54	2617.73	3090287001.10	1575.96	2527.44	3286764535.53
31				1555.32	2491.70	3155971363.79	1517.68	2597.83	3133075122.50
32				1488.79	2738.90	3178637788.39	1538.31	2631.56	3260611907.51
33				1513.31	2651.51	3179434962.43	1545.07	2679.33	3349037869.93
34				1484.57	2537.20	2927900410.13			

35				1490.92	2652.54	3087238630.22			
36				1643.26	2703.35	3822177549.75			

	④			⑤			⑥		
	egg width	egg length	egg volume	egg width	egg length	egg volume	egg width	egg length	egg volume
1	1457.14	2324.45	2584172498.28	1387.88	2647.11	2669769922.65	1460.29	2701.48	3016323923.26
2	1432.76	2466.88	2651521555.12	1385.65	2686.28	2700577984.97	1511.39	2593.37	3101806962.18
3	1503.48	2406.56	2848330935.14	1416.09	2567.47	2695788230.48	1451.56	2610.57	2880063860.05
4	1515.06	2524.86	3034566887.04	1437.31	2543.88	2751683965.86	1456.41	2690.61	2988264994.59
5	1439.03	2591.53	2809940207.09	1380.22	2097.86	2092543421.15	1536.93	2555.06	3160161217.47
6	1431.22	2481.58	2661587139.65	1341.56	2480.62	2337648006.14	1511.91	2697.49	3228587878.50
7	1427.45	2461.26	2625906217.46	1419.83	2510.16	2649540446.19	1438.17	2768.40	2998132359.91
8	1494.17	2536.02	2964517427.53	1398.21	2624.33	2686345822.78	1463.19	2788.89	3126298236.30
9	1435.90	2335.06	2520842011.33	1424.48	2488.26	2643676076.02	1520.01	2596.30	3140831514.70
10	1444.18	2466.06	2693043208.22	1447.48	2511.57	2755282695.85	1497.72	2680.42	3148183453.40
11	1453.50	2591.16	2866309059.01	1420.39	2644.04	2793052677.42	1480.64	2581.05	2962750315.93
12	1422.81	2487.13	2636261125.61	1369.69	2536.02	2491141859.18	1722.58	2637.59	4097909185.36
13	1465.90	2477.27	2787283696.23	1358.56	2584.57	2497734674.13	1460.77	2678.28	2992395849.50
14	1455.87	2406.15	2670335162.99	1453.93	2538.04	2809193983.66	1504.42	2565.27	3039973789.64
15	1429.99	2459.28	2633122540.74	1395.49	2526.95	2576618193.20	1506.30	2656.30	3155709399.82
16	1438.54	2371.45	2569555674.55	1438.67	2586.07	2802589386.24	1475.50	2593.88	2956853186.78

17	1456.23	2449.75	2720078978.73	1393.78	2709.25	2755734382.51	1475.92	2672.70	3048433580.41
18	1386.03	2344.00	2357780965.30	1466.56	2591.53	2918475585.14	1402.50	2582.69	2659957060.34
19	1448.45	2431.74	2671300981.84	1414.90	2531.63	2653710255.23	1454.78	2761.89	3060531651.28
20	1438.11	2450.18	2653281346.36	1476.64	2513.71	2869882205.05	1475.21	2709.74	3087670743.95
21	1479.81	2389.03	2739239326.07	1485.64	2455.40	2837587572.92	1631.45	2749.68	3832018035.02
22	1490.21	2446.87	2845141366.95	1325.67	2258.96	2078636875.05	1508.58	2643.64	3150201303.01
23	1557.87	2440.15	3100823938.58	1369.69	2517.75	2473192458.63	1539.86	2663.97	3307418754.03
24	1503.66	2554.75	3024438553.73	1330.46	2470.03	2289301344.49	1528.64	2671.71	3268866753.47
25	1485.64	2525.77	2918903220.81	1378.94	2636.82	2625260424.93	1493.58	2501.88	2922299301.64
26	1409.09	2413.15	2508767918.48	1404.82	2648.01	2736288169.81	1557.13	2644.44	3357256362.31
27	1416.78	2515.08	2643339017.02	1441.79	2547.55	2772853771.93	1437.62	2713.61	2936540371.91
28	1513.02	2471.17	2962048143.83	1397.26	2661.08	2720271627.82	1546.55	2611.65	3270721049.96
29	1473.47	2440.11	2773893475.47	1462.04	2623.90	2936730728.26	1555.15	2803.89	3550611225.49
30	1461.68	2431.70	2720270939.54	1329.99	2270.22	2102646853.35	1512.79	2704.62	3240866908.36
31	1505.30	2383.89	2828333730.76	1383.55	2610.44	2616370935.97	1697.80	2474.06	3734055410.44
32	1456.23	2513.96	2791372268.42	1443.51	2628.00	2867217429.62	1467.71	2722.48	3070727167.34
33	1467.46	2421.08	2729873589.31	1507.41	2506.22	2981815370.00	1466.80	2636.15	2969697606.04
34	1467.71	2463.59	2778716575.18	1466.56	2549.25	2870860277.16			

35	1550.60	2367.80	2980867571.15	1411.59	2718.07	2835817597.59			
36	1448.63	2405.71	2643379323.26	1350.15	2547.17	2431211960.92			
37	1466.38	2468.71	2779469430.17	1421.81	2617.77	2770863161.07			
38	1433.19	2361.67	2539969459.59	1413.91	2509.49	2626794238.95			
39	1434.24	2605.29	2806073004.70	1382.97	2499.87	2503473746.28			
40	1482.55	2374.98	2733233454.74	1435.66	2285.57	2466573005.94			
41	1475.21	2527.69	2880228333.90						
42	1461.68	2451.52	2742437315.56						
43	1433.93	2558.07	2754025152.34						

Pseudorhodeus tanago (mm)

Female①

	①			②			③		
	egg width	egg length	egg volume	egg width	egg length	egg volume	egg width	egg length	egg volume
1	1476.46	2116.17	2415425927.14	1484.47	2200.13	2538578228.84	1581.27	2307.68	3021240468.87
2	1453.93	1954.96	2163820371.60	1494.79	2253.71	2636670195.89	1626.57	2252.30	3120119870.63
3	1490.39	2028.32	2359034561.14	1572.29	2040.94	2641779678.46	1554.75	2173.92	2751457278.41
4	1462.52	2256.81	2527542942.64	1474.05	2217.51	2522827120.27	1544.21	2193.16	2738304963.27
5	1507.70	2189.94	2606526139.04	1568.63	2192.26	2824420573.77			
6	1496.42	2139.53	2508550413.62	1505.36	2198.99	2609187132.35			
7	1454.78	2163.05	2396936469.14	1541.62	2240.12	2787555391.87			
8	1468.25	2205.97	2489977382.94						
9	1437.31	1986.72	2149013714.20						
10	1490.92	2147.93	2499932772.75						

Female②

	①			②			③		
	egg width	egg length	egg volume	egg width	egg length	egg volume	egg width	egg length	egg volume
1	1525.81	2259.19	2753910584.67	1539.86	2249.75	2793157008.26	1570.51	2351.37	3036710270.64
2	1572.37	2188.69	2833287673.35	1601.30	2156.14	2894805017.03	1628.79	2172.13	3017286406.31
3	1596.55	2235.58	2983693134.37	1608.34	2127.11	2881013303.37	1559.85	2233.41	2845339845.09
4	1585.67	2159.57	2843115853.00	1556.11	2249.60	2852236391.10	1569.56	2154.17	2778645306.21
5	1586.62	2171.31	2861986724.65	1563.08	2115.46	2706224308.07	1586.12	2176.15	2866546526.74
6	1552.19	2207.65	2784963564.74	1485.94	2002.96	2315651069.07	1624.94	2110.07	2917233392.82
7	1559.00	2172.94	2765290360.17	1541.92	2087.74	2598954195.58	1653.75	2248.97	3220501420.28
8	1565.78	2035.32	2612731888.01	1569.16	2058.27	2653606348.80	1583.95	2372.01	3115990878.08
9	1586.12	2240.16	2950864622.49				1608.34	2307.68	3125568312.19

Female③

	①			②			③		
	egg width	egg length	egg volume	egg width	egg length	egg volume	egg width	egg length	egg volume
1	1433.93	2038.31	2194452366.88	1441.79	2444.77	2660983505.31	1497.54	2163.13	2540016600.25
2	1529.68	2418.60	2963202235.31	1344.45	1994.79	1887934062.79	1439.10	2352.69	2551182970.65
3	1396.25	2064.48	2107340692.05	1524.88	2270.07	2763811047.45	1459.14	2291.24	2554247891.54
4	1533.88	2345.21	2889115964.14	1292.49	2159.57	1888954991.94	1404.38	2384.18	2462122176.45
5	1554.69	2245.67	2842065809.80	1511.39	2307.10	2759414386.54	1498.25	2383.26	2801146216.04
6				1429.31	2332.94	2495476927.86	1485.46	2339.29	2702752473.47

Tanakia limbata (mm)

Female①

	①			②			③		
	egg width	egg length	egg volume	egg width	egg length	egg volume	egg width	egg length	egg volume
1	1474.73	2506.22	2853914050.22	1484.57	2649.94	3058001370.40	1491.16	2717.12	3163409220.79
2	1540.95	2484.67	3089185290.11	1413.91	2200.68	2303540874.71	1468.25	2711.86	3060999781.29
3	1541.63	2423.85	3016245881.09	1532.79	2454.22	3019095234.01	1489.44	2663.07	3093338987.58
4	1488.25	2511.43	2912551085.13	1396.82	2341.10	2391656674.70	1461.92	2658.43	2974885721.89
5	1555.83	2439.20	3091509982.27	1504.54	2651.64	3142822170.52	1519.31	2569.22	3105230528.15
6	1577.30	2474.92	3223954062.45	1572.37	2527.27	3271583728.06	1475.21	2544.26	2899110597.14
7	1488.37	2507.17	2908077552.90	1411.59	2155.97	2249372883.22	1518.56	2653.87	3204350464.85
8	1535.04	2484.99	3065919333.17	1526.21	2451.52	2989936617.10	1491.63	2615.40	3046916282.98
9	1500.60	2563.24	3022163086.31	1459.14	2434.60	2714064618.93	1494.17	2678.28	3130811180.89
10	1556.00	2352.91	2982792095.63	1554.75	2319.58	2935821386.99	1471.31	2723.55	3087041820.18
11	1585.79	2317.60	3051590739.21	1508.64	2545.51	3033499308.35	1451.37	2663.20	2937390295.50
12	1543.47	2422.83	3022144342.62	1527.25	2608.81	3186123050.55	1467.83	2686.28	3030389237.87
13	1588.84	2430.10	3212079009.00	1524.65	2541.58	3093443295.61	1495.95	2471.71	2896192697.36
14	1521.17	2491.70	3018905454.19	1496.42	2512.52	2945870298.06	1498.89	2748.14	3232802263.20
15	1507.70	2521.22	3000823534.15	1412.91	2373.64	2481082926.07	1507.35	2608.81	3103635988.98
16	1558.	2401.1	3053055	1547.	2520.4	3158782	1426.	2591.2	2761048

	32	6	429.12	12	1	586.59	53	9	839.04
17	1540.78	2366.60	2941731953.19	1502.25	2559.31	3024155219.55	1467.71	2502.80	2822947853.31
18	1568.04	2431.74	3130606478.05	1474.97	2559.20	2915195193.90	1528.35	2737.45	3348038211.77
19	1394.23	2020.91	2056891531.20	1544.72	2588.94	3234615910.45	1496.42	2727.28	3197668271.79
20	1568.94	2513.96	3240176585.68	1512.32	2531.84	3031958878.20	1451.56	2676.56	2952867113.62
21	1618.79	2519.68	3457200386.84	1508.29	2549.25	3036545752.47	1502.48	2672.07	3158391035.59
22	1551.11	2508.02	3159478669.18	1542.78	2582.83	3218861708.50	1487.90	2614.32	3030430537.11
23	1563.36	2420.60	3097696850.65	1544.21	2533.86	3163691820.87	1469.69	2749.33	3109397314.97
24	1559.85	2439.35	3107705597.08	1527.42	2630.38	3213194852.10	1479.03	2562.51	2935075012.77
25	1596.55	2490.85	3324383320.12	1511.10	2548.87	3047403064.26	1496.77	2586.38	3033908772.57
26	1327.80	2165.74	1999265662.18	1466.26	2585.97	2911020040.71	1402.37	2734.13	2815418472.98
27	1572.76	2413.92	3126413283.75	1542.44	2552.71	3179910156.34	1546.44	2589.55	3242565011.14
28	1566.40	2266.95	2912388511.43	1486.47	2510.87	2904934679.76	1491.63	2602.85	3032291536.49
29	1493.05	2678.31	3126145020.22	1522.62	2517.36	3055815695.48	1506.94	2586.07	3074910603.69
30	1354.72	2167.04	2082411610.83	1535.73	2582.69	3189321943.89	1454.90	2643.34	2929654704.25
31	1546.72	2471.89	3096366581.30	1453.50	2610.74	2887974193.38	1475.92	2632.23	3002270134.36
32	1476.64	2351.71	2684925472.73	1509.52	2533.65	3022885923.27	1493.05	2746.63	3205886365.33
33	1559.00	2509.04	3193007636.96	1517.92	2669.93	3221017359.11	1457.14	2488.79	2766881626.01

34	1557.36	2312.76	2937021505.56	1516.29	2580.06	3105923223.01	1445.03	2498.71	2731930865.14
35	1590.68	2390.36	3166840196.89	1545.35	2586.55	3234262597.28	1485.46	2643.37	3054083870.09
36	1572.76	2488.26	3222699107.98	1465.78	2535.22	2852013391.09	1397.51	2635.85	2695450902.88
37	1570.96	2511.74	3245683556.23	1594.56	2538.00	3378870610.18	1477.90	2688.90	3075117741.53
38	1600.42	2427.78	3255914909.60	1499.84	2574.58	3032440331.32	1442.04	2735.68	2978629392.79
39	1518.56	2425.56	2928676317.03	1525.05	2620.80	3191554203.73	1542.78	2678.01	3337486484.38
40	1581.33	2498.28	3271016351.84				1461.68	2657.60	2972973704.42
41	1529.56	2513.15	3078584300.39				1470.11	2640.43	2987949072.39
42	1539.40	2551.88	3166371897.42				1496.42	2514.10	2947723655.58
43	1545.07	2386.89	2983492554.02				1505.30	2730.77	3239886993.89

Female②

	①			②			③		
	egg width	egg length	egg volume	egg width	egg length	egg volume	egg width	egg length	egg volume
1	1532.27	2499.98	3073310671.26	1629.66	2606.41	3624399681.31	1515.12	2522.62	3032109292.50
2	1546.21	2518.45	3152597853.52	1518.44	2695.73	3254385119.84	1492.34	2523.04	2942109137.85
3	1535.73	2586.48	3194004092.65	1562.85	2578.31	3297378497.73	1485.94	2709.84	3132877514.43
4	1661.00	2429.70	3509860834.09	1524.88	2671.15	3252127405.39	1600.42	2612.36	3503467750.96
5	1639.17	2379.44	3347498296.71	1593.78	2635.51	3505273389.77	1610.48	2481.69	3370203854.18
6	1471.67	2432.97	2759030988.18	1581.33	2657.63	3479651230.53	1531.41	2517.36	3091191475.05
7	1653.33	2331.80	3337396186.20	1556.74	2542.87	3226659624.39	1526.21	2208.56	2693627194.27
8	1511.39	2474.17	2959238288.95	1566.52	2694.35	3461966611.63	1489.74	2689.73	3125541790.30
9	1545.07	2441.56	3051827938.54	1586.84	2613.11	3445275837.69	1549.63	2561.51	3220716238.81
10	1634.48	2496.59	3492223115.59	1570.74	2635.08	3404079754.54	1617.92	2380.92	3263298047.60
11	1626.84	2480.55	3437457130.01	1614.48	2645.78	3610897113.01	1516.29	2434.64	2930864303.80
12	1606.58	2470.03	3338155317.09	1592.62	2549.42	3385822923.39	1521.87	2617.97	3174794432.99
13	1608.72	2500.40	3388219113.89	1573.88	2542.56	3297722778.08	1553.39	2681.57	3388033257.90
14	1674.50	2498.71	3668447669.41	1592.62	2659.26	3531690136.99	1536.13	2618.95	3235785554.22
15	1595.50	2516.77	3354551388.07	1539.17	2614.90	3243596588.36	1516.29	2507.88	3019027009.52
16	1627.71	2507.88	3479033889.71	1595.55	2607.49	3475721148.23	1567.36	2470.85	3178219309.11

17	1513.31	2474.92	2967685180.27	1559.85	2668.50	3399645602.66	1573.38	2455.15	3182311971.84
18	1581.05	2428.79	3178913218.89	1580.32	2501.00	3270415098.22	1476.46	2581.29	2946319948.39
19	1578.53	2480.83	3236704437.36	1586.12	2623.70	3456081279.95	1550.83	2459.14	3096767800.59
20	1636.37	2495.21	3498367493.73	1606.97	2544.78	3440824791.90	1588.01	2693.27	3556192273.68
21	1550.37	2490.39	3134279433.45	1640.84	2555.30	3602234975.70	1529.56	2362.46	2893989549.29
22	1503.36	2567.99	3038920554.96	1533.19	2460.25	3028112741.38	1588.84	2587.75	3420451319.67
23	1584.84	2475.88	3256110027.50	1547.64	2643.74	3315548147.74	1571.58	2399.98	3103701604.42
24	1682.91	2420.64	3589640668.89	1532.96	2584.74	3180383374.53	1594.78	2510.87	3343669420.43
25	1592.84	2451.08	3256126836.36	1568.94	2686.57	3462654311.90	1540.78	2497.93	3104970595.64
26	1624.45	2532.71	3499438319.43	1571.92	2727.66	3528981467.29	1461.92	2538.04	2840167388.76
27	1641.81	2513.92	3548084410.85	1541.63	2709.51	3371728208.23	1548.04	2569.12	3223628677.10
28				1538.31	2731.64	3384621134.10	1571.58	2563.58	3315274628.27
29				1670.54	2554.40	3732509565.38	1556.00	2629.04	3332841460.40
30							1581.27	2538.00	3322790743.80
31							1597.27	2582.80	3450201580.85
32							1623.09	2443.15	3370039531.02
33							1620.32	2561.51	3521237481.98

Female③

	①			②			③		
	egg width	egg length	egg volume	egg width	egg length	egg volume	egg width	egg length	egg volume
1	1563.25	2744.31	3511450764.95	1610.97	2703.05	3673069857.26	1589.29	2657.63	3514786813.49
2	1455.93	2656.43	2948341428.85	1587.57	2618.95	3456121146.43	1539.40	2651.91	3290487759.72
3	1570.01	2741.22	3537908713.81	1522.27	2631.96	3193464906.05	1546.21	2625.58	3286702497.87
4	1565.28	2683.32	3442333583.78	1551.74	2674.32	3371699493.66	1571.58	2675.64	3460190249.35
5	1550.37	2650.28	3335507610.27	1474.19	2728.05	3104250982.04	1447.11	2630.89	2884718398.88
6	1477.06	2610.57	2982156126.27	1595.06	2632.23	3506501610.27	1605.59	2729.99	3684945671.77
7	1528.12	2644.31	3233139616.10	1612.40	2672.24	3637624139.07	1626.84	2734.23	3788992873.22
8	1596.61	2554.40	3409442619.13	1581.77	2521.81	3303690238.66	1535.04	2582.80	3186583161.22
9	1536.13	2608.58	3222973651.87	1592.62	2733.74	3630611807.05	1606.58	2653.24	3585760929.99
10	1607.85	2737.32	3705217127.82	1549.63	2645.78	3326663103.71	1555.32	2745.76	3477763423.31
11	1626.57	2574.51	3566473745.46	1581.77	2809.80	3680971517.88	1621.19	2697.75	3712513130.89
12	1615.90	2569.95	3513581027.89	1581.77	2772.73	3632409073.22	1524.01	2491.38	3029805408.86
13	1569.67	2724.04	3514214623.82	1565.78	2700.47	3466585128.76	1605.26	2819.59	3804318810.29
14	1560.59	2747.79	3503953746.29	1546.55	2509.88	3143264570.73	1615.79	2705.34	3698185328.00
15	1546.55	2565.65	3213103573.01	1661.00	2771.87	4004149017.19	1566.40	2733.10	3511246805.51
16	1676.87	2663.20	3921034405.36	1631.39	2737.61	3814948416.62	1526.90	2700.47	3296563920.79

17	1668.63	2622.28	3822963340.02	1581.77	2765.46	3622886137.27	1518.56	2671.94	3226167128.16
18	1586.68	2739.55	3611223123.20	1625.54	2651.81	3668899096.99	1581.44	2704.52	3541545930.77
19	1569.39	2806.31	3619061330.21	1605.59	2683.28	3621897814.89	1509.98	2561.69	3058228431.86
20	1473.95	2680.16	3048759539.02	1621.19	2710.88	3730573310.80	1524.88	2747.11	3344612114.93
21	1568.88	2650.81	3416317194.73	1533.14	2653.51	3265729806.33	1521.87	2713.25	3290347699.25
22	1604.77	2645.11	3566701570.73	1559.85	2767.28	3525491164.59	1559.85	2706.48	3448025482.07
23	1508.64	2689.10	3204621796.78	1589.29	2653.94	3509906780.92	1568.04	2663.17	3428554126.01
24	1577.02	2741.58	3570048993.96	1644.97	2768.05	3921850437.07	1606.25	2747.79	3712011320.29
25	1621.84	2589.55	3566486338.67	1546.04	2732.32	3419564155.39	1577.24	2683.32	3495174247.76
26	1556.74	2631.99	3339748890.27	1557.02	2670.36	3389661126.62	1522.04	2746.24	3331114607.02
27	1551.57	2767.76	3488741509.15	1527.83	2479.02	3029897774.78	1575.90	2840.77	3693963024.71
28	1605.59	2722.22	3674454453.05	1594.17	2502.84	3330431237.62	1572.54	2748.85	3559182491.08
29	1596.77	2706.12	3612691330.24				1622.49	2698.25	3719175597.98
30	1580.60	2721.22	3559633671.54				1515.35	2680.16	3222457558.92
31	1521.17	2729.77	3307343049.39				1516.69	2699.85	3251874800.59
32	1635.29	2673.00	3742697714.62				1519.54	2734.52	3306019459.44
33	1515.35	2591.70	3116104553.33				1597.93	2674.98	3576313152.06
34	1521.87	2751.51	3336740372.81				1559.29	2672.24	3401934555.49

35	1524.<br>47	2626.9<br>2	3196582<br>949.61						
