# Supplementary figures and images for: Production of offspring via the transplantation of frozen germ cells from Tokyo bitterling, a fish on the brink of extinction
Source: Sci Rep. 2025 Nov 19;15:40759. doi: 10.1038/s41598-025-24449-y (PMC12630933; doi:10.1038/s41598-025-24449-y)

Fig. 3C

*rag1*

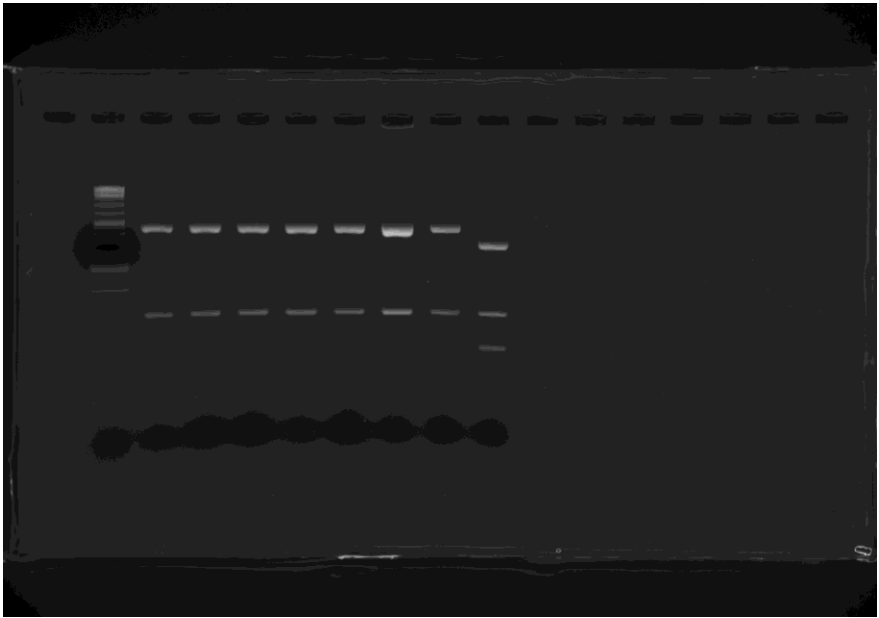

*vasa*

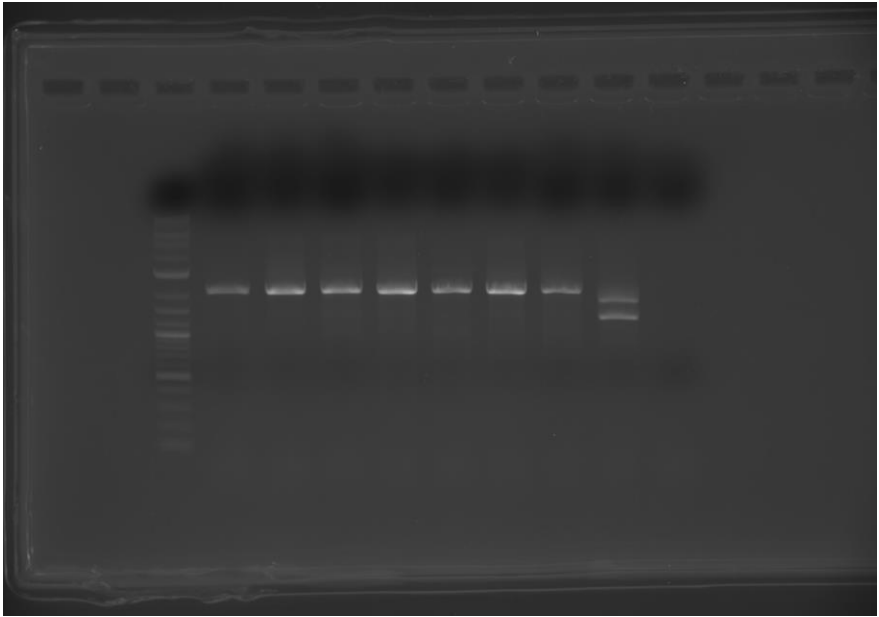

*dnd*

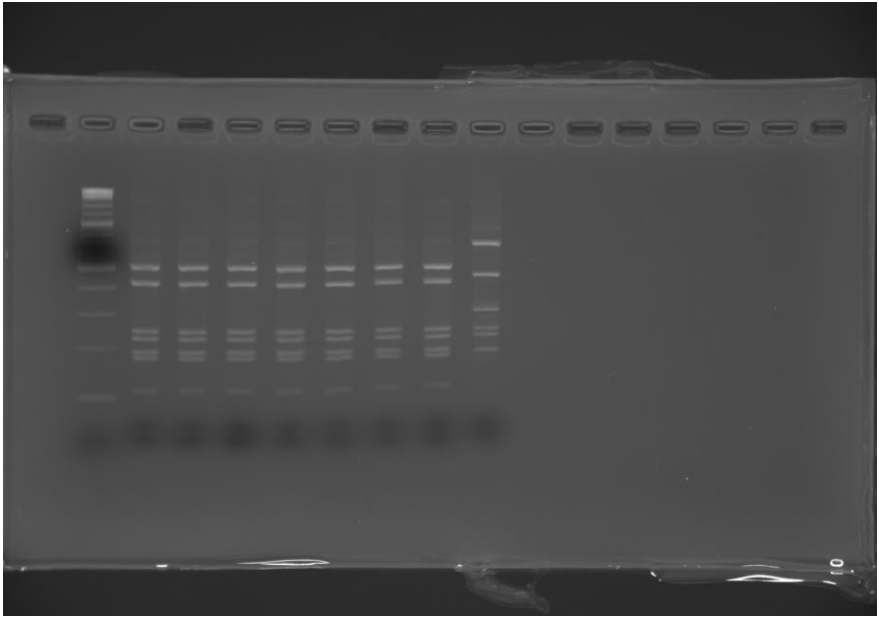

Fig. 4E

*rag1*

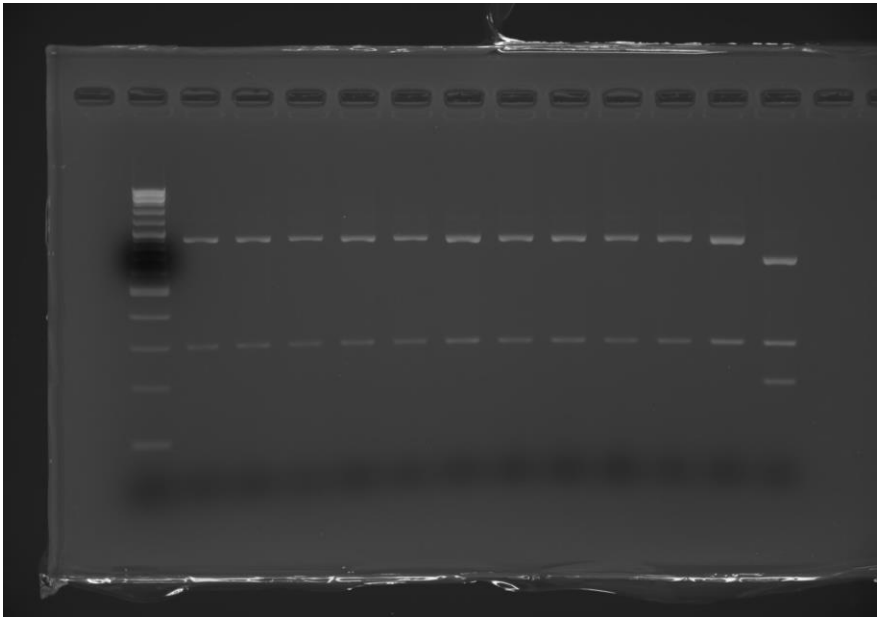

*vasa*

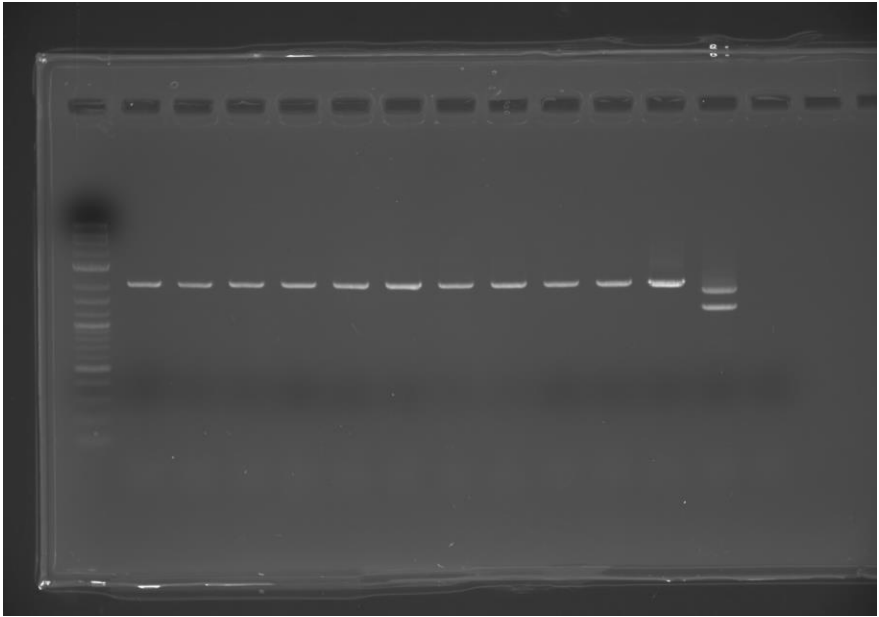

*nd1*

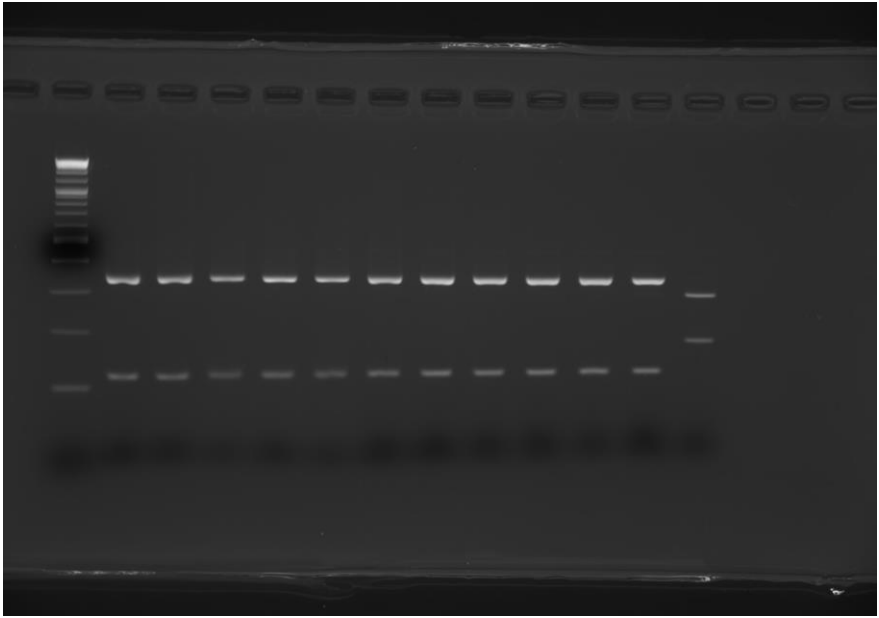

Supplement: Supplementary file 3 — Supplementary Information 3. [file 41598_2025_24449_MOESM3_ESM.pdf]
