# Supplementary material for: Production of offspring via the transplantation of frozen germ cells from Tokyo bitterling, a fish on the brink of extinction
Source: Sci Rep. 2025 Nov 19;15:40759. doi: 10.1038/s41598-025-24449-y (PMC12630933; doi:10.1038/s41598-025-24449-y)
Supplement: Supplementary file 4 — Supplementary Information 4. [file 41598_2025_24449_MOESM4_ESM.docx]

Supplementary table 1. Number of type A spermatogonia (ASG) retained in the testes at each month of age.

| month | SL (mm) | BW (g) | ASG rate (%) | | | testes max size (µm) |
| --- | --- | --- | --- | --- | --- | --- |
|  |  |  | fully immature | slightly mature | mature |  |
| 4 | 27 | 0.476 | 32.4 |  |  | 135.6 |
| 4 | 27 | 0.472 | 24.8 |  |  | 234.9 |
| 4 | 27 | 0.455 | 36.1 |  |  | 262.1 |
| 4 | 26 | 0.341 | 34.5 |  |  | 191.8 |
| 4 | 23 | 0.227 | 42.2 |  |  | 172.4 |
| 5 | 31 | 0.701 |  |  | 4.9 | 948.0 |
| 5 | 31 | 0.654 |  |  | 10.4 | 732.1 |
| 5 | 32 | 0.661 |  | 22.7 |  | 349.0 |
| 5 | 30 | 0.533 |  | 18.6 |  | 348.6 |
| 5 | 24 | 0.276 | 23.4 |  |  | 199.7 |
| 6 | 28.5 | 0.441 | 27.7 |  |  | 235.8 |
| 6 | 26 | 0.349 |  | 17.2 |  | 391.4 |
| 6 | 27.5 | 0.368 |  | 18.3 |  | 648.4 |
| 6 | 26 | 0.297 | 23.4 |  |  | 253.1 |
| 6 | 26 | 0.299 |  | 26.2 |  | 218.5 |
| 7 | 37 | 1.348 |  |  | 8.6 | 560.9 |
| 7 | 33 | 0.743 |  |  | 5.4 | 773.7 |
| 7 | 33 | 0.693 |  | 10.9 |  | 566.9 |
| 7 | 31.5 | 0.647 |  |  | 9.6 | 504.7 |
| 7 | 32.5 | 0.640 |  |  | 13.8 | 502.0 |

SL; standard length

BW; body weight

Supplementary table 2. Early survival of F2 offspring produced by surrogate-derived F1 fish.

| Fertilized date | Parent | | No. of eggs | No. of hatching larvae | Hatching rate (%) |
| --- | --- | --- | --- | --- | --- |
|  | Male | Female |  |  |  |
| 2023.11.22 | Rec. M#1×F#1 | Rec. M#1×F#1 | 3 | 2 | 66.7 |
| 2023.11.29 | Rec. M#1×F#1 | Rec. M#1×F#1 | 9 | 9 | 100.0 |
| 2023.12.06 | Rec. M#1×F#1 | Rec. M#1×F#1 | 15 | 15 | 100.0 |

Rec. M; recipient male

Rec. F; recipient female

Supplementary table 3. Sequences of PCR Primers Used for Each Gene

| gene | Primer name | Primer sequence |
| --- | --- | --- |
| rag1 | rag1_Fw | 5'-CCAGACGATTCCGCTACGATGTG |
|  | rag1_Rv | 5'-CGTCTCTCTCAACAATCTCAGGAACATG |
| vasa | vasa_Fw | 5'-GCAGCGATGAAGGTTGGAGAGGAGGGGAAGG |
|  | vasa_Rv | 5'-GATGGGGATGCCATACTTCTGGACAGGAGT |
| dnd | dnd_F1 | 5'-CAATTTACAGGTGTGCCATCA |
|  | dnd_R1 | 5'-CTGGAAGAGAGGAATCAGAC |
|  | dnd_F2 | 5'-TGGAGGGACAGCATCAGGTT |
|  | dnd_R2 | 5'-TGGAAGAGAGGAATCAGACG |
| ND1 | ND1_F | 5'-CTCCTTCAACCTATTGCAGA |
|  | ND1_R | 5'-AARGGTGCCCGGTTTGTTTC |
